# DNA asymmetry promotes SUMO modification of the single‐stranded DNA‐binding protein RPA

**DOI:** 10.15252/embj.2019103787

**Published:** 2021-09-29

**Authors:** Laurent Cappadocia, Tomasz Kochańczyk, Christopher D Lima

**Affiliations:** ^1^ Structural Biology Program Sloan Kettering Institute New York NY USA; ^2^ Department of Chemistry Université du Québec à Montréal Montréal QC Canada; ^3^ Howard Hughes Medical Institute Sloan Kettering Institute New York NY USA

**Keywords:** E3 ligase, homologous recombination, post‐translational modification, replication protein A, small ubiquitin‐like modifier, DNA Replication, Recombination & Repair, Post-translational Modifications & Proteolysis, Structural Biology

## Abstract

Repair of DNA double‐stranded breaks by homologous recombination (HR) is dependent on DNA end resection and on post‐translational modification of repair factors. In budding yeast, single‐stranded DNA is coated by replication protein A (RPA) following DNA end resection, and DNA–RPA complexes are then SUMO‐modified by the E3 ligase Siz2 to promote repair. Here, we show using enzymatic assays that DNA duplexes containing 3' single‐stranded DNA overhangs increase the rate of RPA SUMO modification by Siz2. The SAP domain of Siz2 binds DNA duplexes and makes a key contribution to this process as highlighted by models and a crystal structure of Siz2 and by assays performed using protein mutants. Enzymatic assays performed using DNA that can accommodate multiple RPA proteins suggest a model in which the SUMO‐RPA signal is amplified by successive rounds of Siz2‐dependent SUMO modification of RPA and dissociation of SUMO‐RPA at the junction between single‐ and double‐stranded DNA. Our results provide insights on how DNA architecture scaffolds a substrate and E3 ligase to promote SUMO modification in the context of DNA repair.

## Graphical abstract

Structural and biochemical studies of the yeast SUMO E3 ligase Siz2 offer insight into the molecular basis of DNA damage‐triggered SUMO group modification.

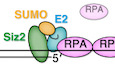

## Introduction

**Figure 1:**
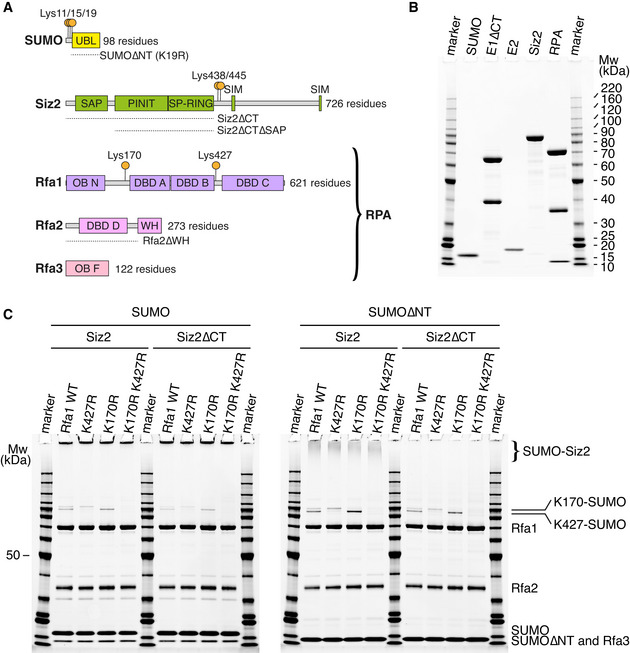
RPA is SUMO modified on two major sites Diagrams showing the domain organization of SUMO (Smt3), Siz2, Rfa1, Rfa2, and Rfa3. Orange circle indicates known (Rfa1 and Smt3) or predicted (Siz2) SUMO modification sites. Dash lines represent truncation mutants used in this study in addition to the full‐length proteins.SYPRO‐stained SDS–PAGE showing purified recombinant SUMO, E1∆CT, E2, Siz2, and RPA proteins. Mass in kilodaltons is annotated for all bands for the molecular weight marker.SUMO conjugation assays performed under multiple‐turnover conditions using wild‐type or mutant forms of RPA, SUMO, and Siz2. Only the 80‐min time points are presented to highlight the formation of Rfa1‐SUMO and Siz2‐SUMO species. The complete time series are presented in Appendix Fig S1. Bands for Mw marker are fully annotated in Fig 1B.

Maintaining genome stability requires that DNA lesions such as double‐stranded breaks are repaired quickly and with high fidelity to prevent toxicity and cell death. In eukaryotes, homologous recombination (HR) constitutes a major pathway to repair damaged DNA (Krejci *et al*, 2012). After a DNA double‐stranded break is formed, the 5' DNA strand is resected to generate DNA duplexes (dsDNA) with long 3' single‐stranded DNA (ssDNA) overhangs that can span up to several kilobases (Chung *et al*, 2010). The resulting ssDNA is coated by the heterotrimeric ssDNA‐binding protein replication protein A (RPA) (Fig 1A), presumably to protect the ssDNA from further insults or to prevent nonspecific recombination. In turn, RPA‐coated ssDNA generates a platform that can recruit DNA repair factors (Park *et al*, 1996; Zou & Elledge, 2003; Davies *et al*, 2008; Dou *et al*, 2010; Maréchal & Zou, 2015) to facilitate DNA repair through one of several HR sub‐pathways (Krejci *et al*, 2012).

Proteins involved in HR are targeted and regulated by different post‐translational modifications, from phosphorylation to conjugation to ubiquitin‐like proteins such as SUMO (Maréchal & Zou, 2015; Zilio *et al*, 2017). SUMO is structurally related to ubiquitin and can be conjugated to lysine residues of substrate proteins by the sequential activities of an E1 activating enzyme, an E2 conjugating enzyme and E3 ligases that activate the E2 for discharge and provide specificity for particular substrates (reviewed in Cappadocia & Lima, 2018). SUMO can promote signaling through non‐covalent interactions with other proteins that contain SUMO‐interacting motifs (SIM) (Cappadocia & Lima, 2018). In budding yeast and humans, induction of DNA damage, including DNA double‐stranded breaks, results in SUMO modification of numerous repair factors (Psakhye & Jentsch, 2012; Yin *et al*, 2012; Hendriks & Vertegaal, 2015; Hendriks *et al*, 2015), including RPA (Burgess *et al*, 2007; Cremona *et al*, 2012; Psakhye & Jentsch, 2012; Bhagwat *et al*, 2021; Charifi *et al*, 2021), in a process termed protein group modification (Psakhye & Jentsch, 2012; Jentsch & Psakhye, 2013). SUMO protein group modification during DNA repair is believed to occur on DNA (Psakhye & Jentsch, 2012) as numerous SUMO substrates and SUMO E3s bind DNA directly (Parker *et al*, 2008; Suzuki *et al*, 2009; Psakhye & Jentsch, 2012; Ulrich, 2014; Varejao *et al*, 2018). In budding yeast, the response to genotoxic stress is triggered by the formation of single‐stranded DNA and leads to protein group modification by SUMO in a manner that is dependent on the SUMO E3 ligase Siz2 (Cremona *et al*, 2012; Psakhye & Jentsch, 2012). Group modification of proteins involved in HR contributes to DNA repair, possibly by forming efficient repair foci by reinforcing interactions between DNA repair proteins through SUMO–SIM interactions as many DNA repair proteins also contain SIMs (Psakhye & Jentsch, 2012; Jentsch & Psakhye, 2013; Dhingra *et al*, 2019). In the case of RPA, its modification by SUMO has been reported to lead to an increased interaction with the checkpoint adapter Sgs1 (Dhingra *et al*, 2019), a DNA helicase that contains four SIMs (Bonner *et al*, 2016). In turn, the increased interaction between Sgs1 and RPA positively contributes to DNA repair by enhancing the DNA damage checkpoint response (Dhingra *et al*, 2019).

In budding yeast, RPA is conjugated to SUMO in a Siz2‐dependent manner upon exposure to DNA damaging agents (Psakhye & Jentsch, 2012; Chung & Zhao, 2015; Dhingra *et al*, 2019). Mass spectrometry and genetic studies revealed that SUMO modification of RPA occurs at two major sites on the Rfa1 subunit, lysine 170 and lysine 427 (Psakhye & Jentsch, 2012; Dhingra *et al*, 2019) (Fig 1A) that account for 75% of Rfa1‐SUMO modification under DNA stress conditions (Dhingra *et al*, 2019). Further analysis revealed that Siz2 and RPA can interact through contacts between the Rfa2 subunit WH domain and the SAP domain of Siz2 (Chung & Zhao, 2015). While Siz2 and RPA can bind DNA to co‐localize the E3 and substrate, it remains unclear whether DNA topology contributes to Siz2‐mediated SUMO conjugation of RPA as proposed in other systems where DNA has been shown to modulate SUMO conjugation either by acting on the substrate or by acting on the SUMO E3. For instance, SUMO modification of the Rpc53 subunit of the RNA Pol III is dependent on the binding of the SUMO E3 ligase Siz1 to DNA (Wang *et al*, 2018) while SUMO conjugation to PCNA is enhanced by its binding to DNA (Parker *et al*, 2008). In this latter instance, enhanced SUMO modification appears independent of Siz1’s ability to bind DNA. Recent evidence also suggests that DNA stimulates the Mms21 SUMO E3 ligase in the context of the Smc5/6 complex (Varejao *et al*, 2018) and that binding of the Smc5/6 complex to collapsed forks triggers Mms21‐dependent SUMO conjugation to fork‐associated DNA repair proteins (Whalen *et al*, 2020).

To determine the contributions of DNA architecture to Siz2‐mediated SUMO conjugation of RPA, we reconstituted SUMO modification of RPA in the presence of different DNA structures. We show that a DNA duplex containing a 3' ssDNA overhang but not a 5' ssDNA overhang stimulates Siz2‐mediated SUMO conjugation of RPA. This result is consistent with the topology of resected DNA ends *in vivo*. Subsequent mutational analysis reveals that protein–DNA and protein–protein interactions contribute to Siz2‐dependent RPA modification. Finally, we propose that the SUMO‐RPA signal can be amplified because SUMO‐RPA complexes readily exchange with RPA to enable successive rounds of Siz2‐dependent SUMO modification of RPA in a manner that is dependent on the resected DNA end.

## Results

### Siz2 promotes SUMO modification of RPA on two major sites

SUMO, E1, E2, Siz2, and RPA were expressed and purified using a bacterial system (Fig 1A and B). SUMO conjugation assays were then employed under multiple‐turnover conditions to characterize Siz2‐dependent SUMO conjugation of RPA (Fig 1C). Analysis by gel electrophoresis reveals that Rfa1 is the principle target of SUMO modification within the RPA complex with two dominant sites as evidenced by time‐dependent accumulation of two bands migrating slower than Rfa1 (Fig 1C and Appendix Fig S1). Consistent with *in vivo* data (Psakhye & Jentsch, 2012; Dhingra *et al*, 2019) (Fig 1A), mutational analysis suggests that SUMO conjugation occurs on Rfa1 lysine 170 (top band) and lysine 427 (bottom band). Concurrent with formation of SUMO‐RPA, we noted the rapid accumulation of high‐molecular‐weight protein species that migrate near the top of the gel. These species do not contain RPA (Fig 1, Appendix Fig S2) and likely correspond to multi‐ and/or poly‐SUMO‐Siz2 proteins as both SUMO and Siz2 possess multiple SUMO conjugation sites (Bylebyl *et al*, 2003; Takahashi *et al*, 2003) (Fig 1A). As these species decrease or alter Siz2 activities or complicate quantitation of SUMO‐RPA, we produced SUMO and Siz2 variants to remove SUMO modification sites within their N‐ and C‐terminal regions (Bylebyl *et al*, 2003; Takahashi *et al*, 2003) (Fig 1A), respectively. Reactions containing Siz2∆CT and SUMO did not decrease the amount of high‐molecular‐weight species but combining Siz2 and SUMO∆NT or Siz2∆CT and SUMO∆NT diminished or nearly eliminated production of these species, respectively (Fig 1C, Appendix Figs S1 and S2). Importantly, the use of these Siz2 and SUMO variants did not appear to alter specificity for modification of Rfa1 on Lys170 and Lys427, so these variants were used to further characterize SUMO conjugation to RPA.

### Siz2 exhibits a preference for RPA bound to a DNA duplex with a 3'‐overhang

**Figure 2:**
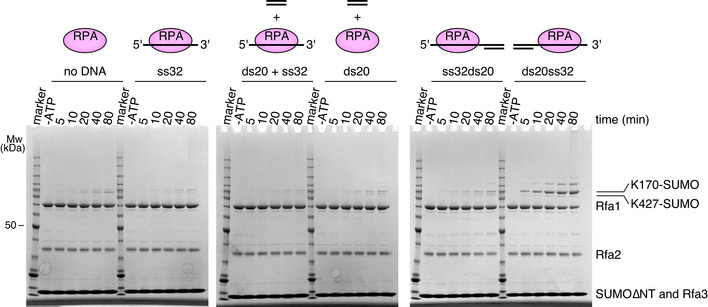
DNA with a 3' overhang stimulates SUMO modification of RPA by Siz2 SUMO conjugation assays performed under multiple‐turnover conditions using Siz2∆CT and different RPA‐DNA complexes. SDS–PAGE were stained with Coomassie. Bands for Mw marker are fully annotated in Fig 1B.

Siz2 binds double‐stranded DNA (Psakhye & Jentsch, 2012) while RPA binds single‐stranded DNA (Wold & Kelly, 1988), however neither exhibits much sequence specificity (Kim *et al*, 1992; Suzuki *et al*, 2009; Psakhye & Jentsch, 2012). To understand whether DNA architecture contributes to SUMO conjugation of RPA, we performed conjugation reactions under multiple‐turnover conditions using DNA structures containing double‐stranded and/or single‐stranded DNA. Most DNA structures had a negligible effect on SUMO conjugation to RPA (Fig 2), but a complex between RPA and a DNA duplex with a 3'‐overhang led to increased RPA modification by SUMO on residue 170 of the Rfa1 subunit (rightmost panel in Fig 2). While an increase in SUMO modification of RPA was dependent on the presence of a DNA duplex with a 3'‐overhang, it was independent of a 5'‐phosphate end at the junction (Appendix Fig S3A and B). Overall, these results underscore that enhanced modification of RPA is dependent on a 3'‐overhang DNA substrate, an architecture analogous to that formed after resection prior to DNA double‐stranded break repair (Krogh & Symington, 2004; Orans *et al*, 2011). Consistent with this model, SUMO conjugation to RPA was enhanced to similar degrees for all other DNA structures tested as long as they contain a DNA duplex adjacent to a 3'‐ssDNA overhang (Appendix Fig S3C and D). While the specific site of SUMO modification is not relevant for function *in vivo* (Psakhye & Jentsch, 2012), modification on lysine 170 is increased in the presence of a DNA duplex with a 3'‐overhang while SUMO modification of RPA on lysine 427 of Rfa1 is diminished in conditions containing DNA, perhaps consistent with the analogous residues being partially buried in the DNA interface in a structure of RPA from *U. maydis* in complex with single‐stranded DNA along with the polarity of ssDNA in this structure (Fan & Pavletich, 2012). Overall, these results suggest that the DNA structure formed after DNA resection can facilitate SUMO modification of RPA by Siz2.

### DNA duplex with a 3'‐overhang increases the rate of SUMO conjugation to RPA

**Figure 3:**
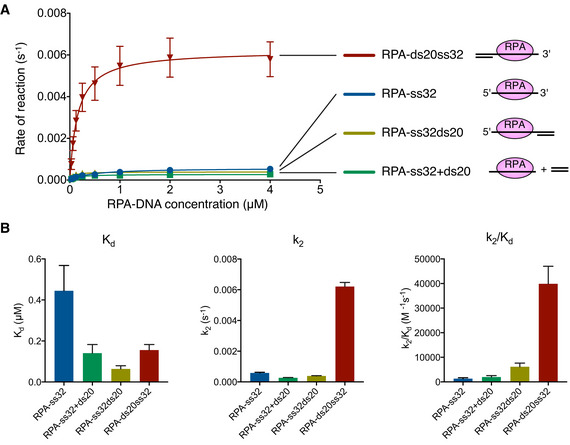
DNA with a 3' overhang increases SUMO conjugation to RPA Plot showing the initial rate of SUMO conjugation versus RPA‐DNA concentration as obtained from single‐turnover reactions to measure rates of SUMO conjugation to Rfa1 with different RPA–DNA complexes. Representative gels are shown in Appendix Fig S4.Histograms presenting *K*_d_, k_2_, and specificity (k_2_/*K*_d_) constant for SUMO conjugation to RPA‐DNA. Data information: In (A), data show mean ± SD of three technical replicates. In (B), histograms show mean ± SEM of the three technical replicates presented in (A).

To quantify the contribution of DNA architecture to SUMO modification of RPA, we determined the rates and binding constants for the four different RPA/DNA complexes using single‐turnover conditions (Fig 3A and B, Appendix Fig S4). While comparable dissociation constants were observed for each reaction containing duplex DNA, a ten‐fold higher rate (k_2_) was observed for the RPA‐ds20ss32 complex. This suggests that a DNA duplex with a 3'‐overhang does not increase SUMO modification of Rfa1 through increased association, but rather that duplex DNA with a 3' overhang increases the rate of SUMO modification on lysine 170. We envision that this is due to productive positioning of the SUMO conjugation apparatus proximal to Rfa1 lysine 170.

### SUMO modification of RPA requires the Siz2 SAP domain and is partially dependent on Rfa2 WH domain

**Figure 4:**
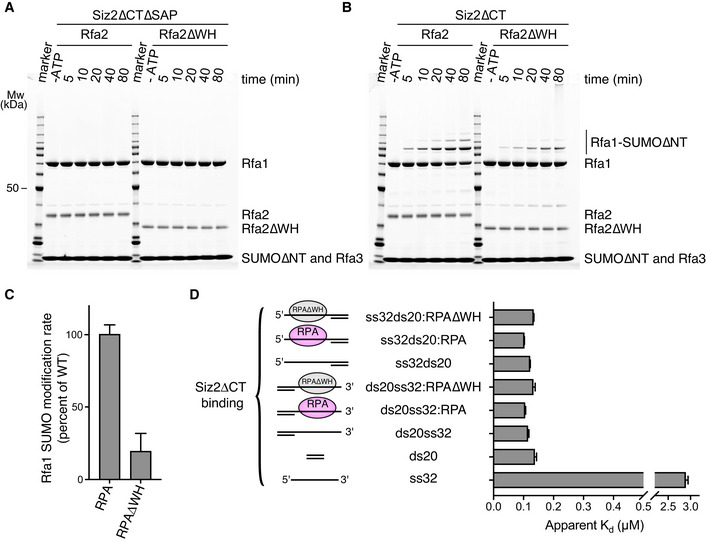
SUMO conjugation to RPA is dependent on Siz2 SAP domain and partially dependent on Rfa2 WH domain A, BSUMO conjugation assays performed under multiple‐turnover conditions using RPA complexes with or without the WH domain of Rfa2 in the presence of Siz2∆CT lacking (A) or including (B) its SAP domain. In both cases, reactions were done in technical triplicate and SDS–PAGE were stained with SYPRO. Only one representative gel is shown. Bands for Mw marker are fully annotated in Fig 1B.CHistograms derived from the gels presented in (B) and presenting Rfa1‐SUMO conjugation rates for RPA complexes with or without the WH domain of Rfa2.DHistograms derived from the curves presented in Appendix Fig S5 and presenting apparent dissociation constants (*K*_d_) of Siz2∆CT with DNA and its complexes with RPA or RPA∆WH. Data information: In (C) and in (D), data show mean ± SD for three technical replicates.

Prior biochemical and genetic experiments in yeast suggested a model wherein the SAP domain of Siz2 interacted directly with RPA through contacts to the winged helix (WH) domain of the Rfa2 subunit (Chung & Zhao, 2015). As the SAP domain of Siz2 is exclusively composed of α‐helices (Suzuki *et al*, 2009), its interaction with the WH domain of RPA could be similar to the ones of UNG2 (Mer *et al*, 2000) or SMARCAL1 (Feldkamp *et al*, 2014). To determine the impact on SUMO conjugation of RPA after deleting either the SAP domain of Siz2 or the WH domain of Rfa2, conjugation assays were conducted under multiple‐turnover conditions using Siz2 and Rfa2 variants (Fig 4A–C). These data reveal the importance of the Siz2 SAP domain as its deletion resulted in no detectable SUMO conjugation to RPA. In contrast, deletion of the Rfa2 WH domain only led to a 5‐fold decrease in SUMO conjugation to RPA. Importantly, deletion of the WH domain of Rfa2 did not alter the binding of Siz2 to DNA and fluorescence polarization experiments reveal that Siz2 binds DNA‐bound wild‐type RPA and RPA lacking the WH domain of Rfa2 with comparable affinity (Fig 4D and Appendix Fig S5). While the Rfa2 WH domain contributes to SUMO conjugation of RPA, these data underscore the importance of the Siz2 SAP domain with respect to its roles in DNA binding.

### The PINIT and SP‐RING domains of Siz2 are structurally similar to those of Siz1

A fragment of Siz2 encompassing the PINIT and SP‐RING domains is inactive toward RPA (Fig 4A). This was unexpected because an equivalent fragment of Siz1 retains activity and specificity toward substrates such as PCNA *in vitro* (Yunus & Lima, 2009b). We queried if this fragment of Siz2 differs in any substantive manner from Siz1 relative to its substrate, E2 or SUMO binding surfaces by obtaining a crystal structure of this fragment at a resolution of 2.65 Å (Appendix Table S1). The structure includes two protomers in the asymmetric unit that share high structural similarity (0.7 Å rmsd over 261 Cα atoms). Protomer A exhibits slightly better densities and is depicted in related figures.

**Figure 5:**
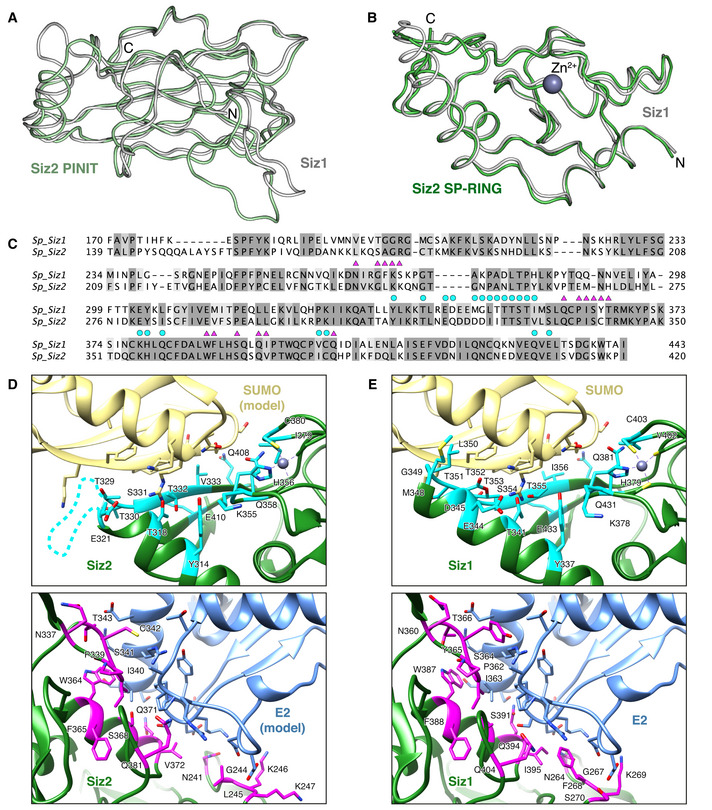
Residues that interact with E2˜SUMO are mostly conserved between Siz2 and Siz1 A, BStructural comparison of Siz2 (green) and Siz1 (white; pdb 5JNE) highlighting the structural similarity of (A) the PINIT domain or (B) the SP‐RING domain.CStructure‐based amino acid alignment of Siz1 and Siz2 PINIT and SP‐RING domains. Residues in Siz1 that make contacts within 4 Å of SUMO and E2 in the PCNA/Siz1/Ubc9˜SUMO complex (pdb 5JNE) are indicated by cyan circles and magenta triangles shown above the sequence, respectively. Contacts determined using the CCP4 program CONTACT (Winn *et al*, 2011).D, EPutative Siz2 E2˜SUMO interaction interface modeled by aligning PINIT and SP‐RING domains of Siz2 to those of Siz1 in the context of the PCNA/Siz1/Ubc9˜SUMO complex (pdb 5JNE) (Streich & Lima, 2016) (D) and Siz1 E2˜SUMO interactions present in the PCNA/Siz1/Ubc9˜SUMO complex (E). Contact forming residues are shown in stick representation and Siz1/Siz2 residues that make contacts with SUMO and E2 are additionally colored in cyan and magenta, respectively.

The structure of Siz2 includes the N‐terminal PINIT domain as well as the SP‐RING and SP‐CTD domains as described previously for structures of Siz1 (Streich & Lima, 2016). The structures of Siz1 and Siz2 can be superposed with an RMSD of 2.2 Å rmsd over 256 Cα atoms. The largest overall deviations arise due to differing orientations of the PINIT domains relative to the SP‐RING domains and to different conformations of the 210‐220 loop within the PINIT domain, so if the PINIT and SP‐RING domains are aligned separately without the 210‐220 loop, they superpose with a 1.5 Å rmsd over 124 Cα atoms–sequence identity of 41% (Fig 5A) or a 1.0 Å rmsd over 123 Cα atoms–sequence identity of 63% (Fig 5B), respectively.

The amino acid residues that are important for contacting the E2 and SUMO in Siz1 are generally well conserved between Siz2 and Siz1 (Fig 5C–E). Residues that are not strictly conserved still retain compensating interactions that are predicted to mediate contacts between the E2 and SUMO. The Siz2 structure suggests that its PINIT and SP‐RING domain are correctly folded, with the latter appearing competent for E2 and SUMO binding. These observations, combined with our inability to detect activities for this fragment, underscore a role for dsDNA, the SAP domain, and ssDNA in templating a complex to promote RPA binding and modification. This is consistent with *in vivo* findings where SAP mutation or replacement with a different type of DNA‐binding domain leads to reduced SUMO modification of RPA under DNA stress conditions (Psakhye & Jentsch, 2012; Chung & Zhao, 2015).

### Mutations within the SAP domain alter SUMO conjugation to RPA

**Figure 6:**
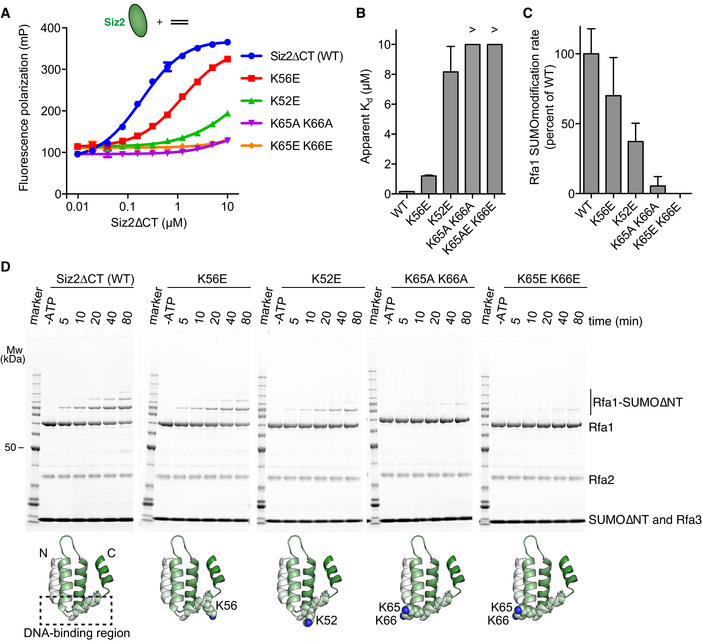
Siz2 DNA‐binding activity is correlated with SUMO conjugation to RPA *in vitro* Fluorescence polarization assays performed with 50 nM 6‐FAM‐ds20, a 6‐FAM‐labeled 20 nucleotide double‐stranded DNA, and serially diluted Siz2∆CT or Siz2∆CT mutant.Histograms derived from the curves presented in (A) and presenting apparent dissociation constants (*K*_d_) for each Siz2∆CT variant.Histograms derived from the gels presented in (D) and presenting Rfa1‐SUMO conjugation rates for RPA complexes in the presence of different Siz2∆CT variants.SUMO conjugation assays performed under multiple‐turnover conditions as in Fig 4. Experiments were performed in technical triplicates (six times for WT). The structural models highlight the position of the mutations using a homology model of the SAP domain of Siz2 obtained using the structure of Siz1 (PDB 2RNN) as a template. A dashed box indicates the relative position of residues that undergo chemical shift perturbation upon DNA binding according to Suzuki *et al* (2009). Bands for Mw marker are fully annotated in Fig 1B. Data information: In (A), data show mean ± SD for three technical replicates. Data were fitted to a single‐site binding model accounting for ligand depletion and no detectable binding was measured for Siz2∆CT K65E K66E. In (B), data show mean ± SEM for three technical replicates presented in (A). In (C), data show mean ± SD for three technical replicates (six for WT).

Alanine and charge‐reversal substitutions were generated at various Siz2 positions within the SAP domain in an attempt to understand whether specific Siz2 residues contribute to DNA binding and SUMO modification of RPA. Each mutant was assessed for its ability to bind a fluorescein‐labeled 20‐mer dsDNA and for its ability to catalyze SUMO modification of RPA when bound to a 20‐mer dsDNA with a 32 nucleotide 3' overhang. Four lysine residues (K52, K56, K65, and K66) were selected because of their predicted proximity to DNA based on a solution structure of the related Siz1 SAP domain as the equivalent positions undergo chemical shift perturbations after addition of dsDNA (Suzuki *et al*, 2009). Consistent with predictions, we found that mutations at positions 52, 56, 65, and 66 diminished DNA‐binding as measured by fluorescence polarization with the most dramatic effect obtained when mutations were simultaneously introduced at positions 65 and 66 (Fig 6A and B). SUMO conjugation to RPA was also impaired for each of these mutants in a manner that appears proportional to the measured DNA‐binding defects (Fig 6C and D).

**Figure 7:**
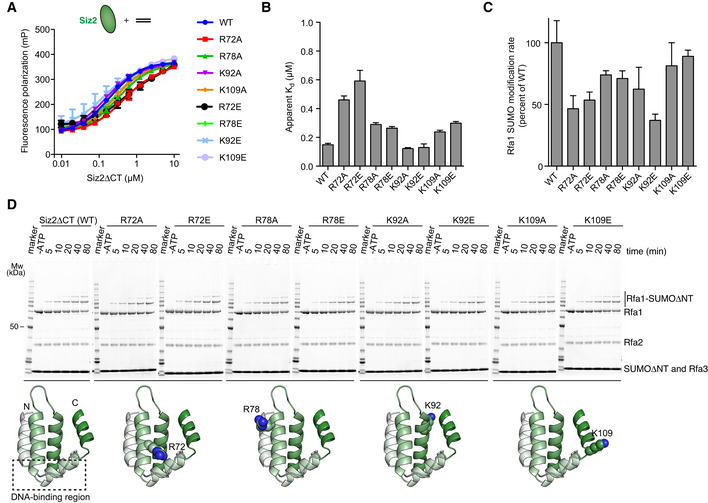
Some residues that differ between Siz2 and Siz1 contribute to SUMO conjugation of RPA Fluorescence polarization assays performed with 50 nM 6‐FAM‐ds20 and serially diluted Siz2∆CT or Siz2∆CT mutant.Histograms derived from the curves presented in (A) and presenting apparent dissociation constants (*K*_d_) for each Siz2∆CT variant.Histograms derived from the gels presented in (D) and presenting Rfa1‐SUMO conjugation rates for RPA complexes in the presence of different Siz2∆CT variants.SUMO conjugation assays performed under multiple‐turnover conditions as in Fig 4 and using different Siz2∆CT variants. The structural models highlight the position of the mutations using a homology model of the SAP domain of Siz2 obtained using the structure of Siz1 (PDB 2RNN) as a template. A dashed box indicates the relative position of residues that undergo chemical shift perturbation upon DNA binding according to Suzuki *et al* (2009). Bands for Mw marker are fully annotated in Fig 1B. Data information: In (A), data show mean ± SD for three technical replicates. Data were fitted to a single‐site binding model accounting for ligand depletion. In (B), data show mean ± SEM for three technical replicates presented in (A). In (C), data show mean ± SD for three technical replicates except for WT. The values for WT (six technical replicates) are the same as in Fig 6 (these experiments were all performed at the same time).

Simultaneous introduction of alanine or charge‐reversal mutations at six residue positions where the SAP domains of Siz1 and Siz2 differ decreased SUMO modification of RPA *in vivo* (Chung & Zhao, 2015). We substituted residues at four of these positions (R72, R78, K92, and K109) and report that substitution to either alanine or glutamate at each of the four positions does not disrupt DNA‐binding (Fig 7A and B) or SUMO conjugation to RPA (Fig 7C and D). Collectively, these results underscore the importance of SAP‐dependent DNA binding.

### SUMO‐RPA binds ssDNA but is more readily displaced from ssDNA than RPA

**Figure 8:**
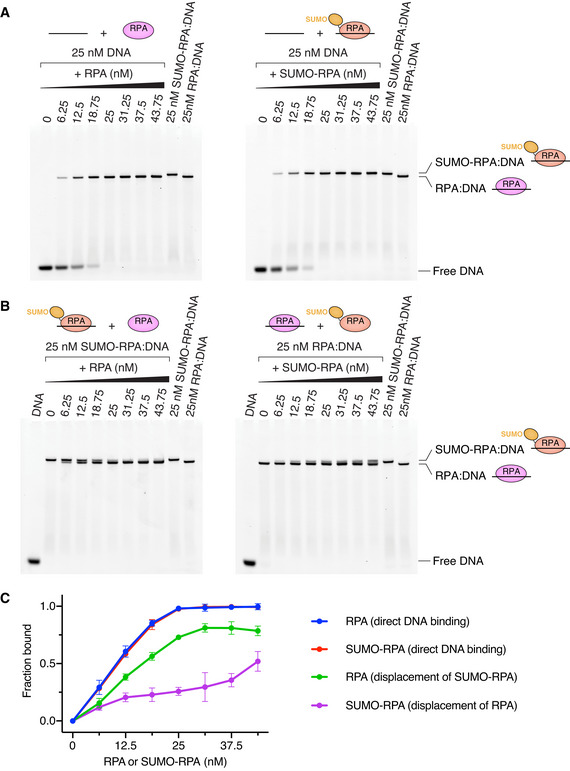
RPA and SUMO‐RPA exchange on ssDNA A, BElectrophoretic mobility shift assays (EMSA) to assess binding of RPA or SUMO‐RPA to 25 nM single‐stranded 6‐FAM‐labeled 32dT DNA and displacement of SUMO‐RPA from 25 nM SUMO‐RPA bound to single‐stranded 6‐FAM‐labeled 32dT DNA by RPA and *vice versa*. All titrations were done in technical triplicates. One representative gel is shown.CData derived from the gels presented in (A) and (B) presenting binding of RPA and SUMO‐RPA to DNA in binding and displacement conditions. Data information: In (C), data show mean ± SD for three technical replicates.

**Figure 9:**
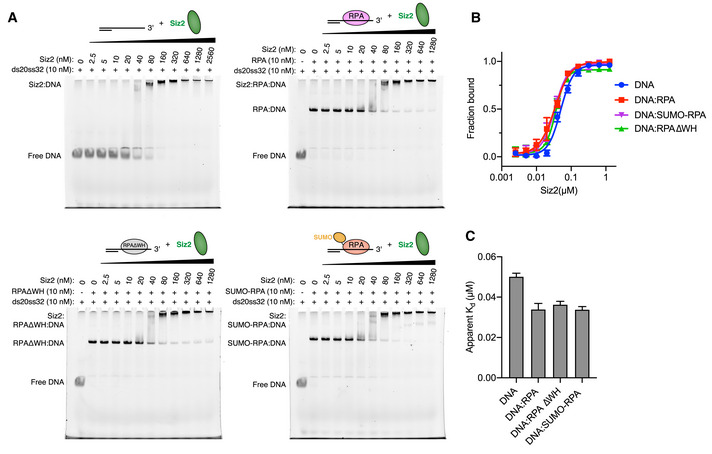
Binding of full‐length Siz2 to ds20ss32 DNA or ds20ss32‐RPA complexes Electrophoretic mobility shift assays (EMSA) performed with 10 nM 6‐FAM‐ds20ss32, a 6‐FAM‐labeled 20‐mer dsDNA with a 32 nucleotide 3' overhang and its complexes with RPA, RPA with K170‐SUMO‐modified Rfa1 or RPA reconstituted with ∆WH‐Rfa2 and serially diluted Siz2. All titrations were done in technical triplicates. One representative gel is shown.Binding curves derived from the gels presented in (A) and presenting Siz2 binding to DNA and DNA–RPA complexes with K170‐SUMO‐modified Rfa1 or without the WH domain of Rfa2.Histograms derived from the curves presented in (B) and presenting apparent dissociation constants (*K*_d_) for Siz2 binding to DNA and DNA–RPA complexes with RPA with K170‐SUMO‐modified Rfa1 or RPA reconstituted with ∆WH‐Rfa2. Data information: In (B) data show mean ± SD for three technical replicates. In (C) data show mean ± SEM for three technical replicates presented in (B).

RPA is highly dynamic and readily exchanges on ssDNA (Ma *et al*, 2017), but it is not clear whether SUMO‐RPA is endowed with similar properties. To determine whether SUMO‐conjugated RPA alters its ssDNA‐binding activity, SUMO‐conjugated RPA (SUMO‐RPA; Appendix Fig S6A) was purified and compared to unmodified RPA with respect to ssDNA‐binding activity. Both RPA and SUMO‐RPA bind ssDNA with sub‐nanomolar affinity as determined by fluorescence polarization experiments (Appendix Fig S6B). While binding activities of RPA and SUMO‐RPA to ssDNA appeared indistinguishable by electrophoretic mobility gel shift assay (Fig 8A), we observed that RPA more readily displaces DNA‐bound SUMO‐RPA compared with SUMO‐RPA displacement of DNA‐bound RPA (Fig 8B and C). We also tested whether SUMO‐conjugated RPA influenced its interactions with Siz2 by performing electrophoretic mobility shift assays using full‐length Siz2 that contains two SIMs that could potentially interact with SUMO‐RPA (Fig 9A–C). These experiments reveal that full‐length Siz2 interacts with DNA‐bound RPA and DNA‐bound SUMO‐RPA with comparable affinity. Overall, these data suggest that SUMO‐RPA binds ssDNA, but is more readily displaced from ssDNA than unmodified RPA and that SUMO‐conjugated RPA does not further enhance interaction with Siz2.

### Amplification of the SUMO‐RPA signal

**Figure 10:**
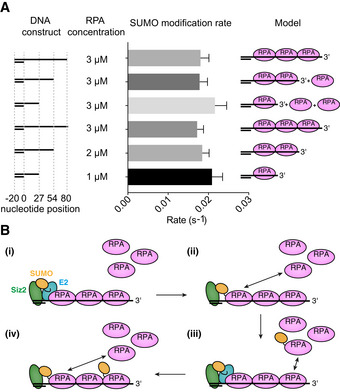
SUMO modification of RPA appears independent of ssDNA length Histograms depicting SUMO conjugation rates to Rfa1 for different RPA–DNA complexes. Siz2 and DNA are both present in the reaction at a concentration of 1 µM while RPA concentration is varied from 1 to 3 µM and the length of single‐stranded DNA is varied from 27 to 80 nucleotides. Representative gels are shown in Appendix Fig S7.A model for SUMO signal amplification. (i) Siz2 binds dsDNA and modifies with SUMO the RPA that is closest to the dsDNA/ssDNA junction. (ii) SUMO‐modified RPA is exchanged with an unmodified RPA. (iii) Siz2 modifies the RPA that is closest to the dsDNA/ssDNA junction. (iv) SUMO‐modified RPA is exchanged with an unmodified RPA. Data information: In (A), data show mean ± SEM of three technical replicates.

Our results thus far suggest that a dsDNA with a 3'‐overhang promotes Siz2‐dependent SUMO conjugation to RPA and that SUMO‐conjugated RPA is more readily displaced from ssDNA by RPA. In this model, we envision that the Siz2 SAP domain binds dsDNA to position it proximal to RPA that is bound to the 3' ssDNA. Once modified, SUMO‐RPA is displaced by RPA to repeat the cycle of conjugation and displacement. Our substrates thus far include only enough ssDNA for one RPA per DNA substrate, but RPA coats much longer ssDNA 3' overhangs in DNA repair (Chung *et al*, 2010). To test the modification and exchange model, SUMO conjugation assays were performed using DNA substrates that contained a 20 nt dsDNA segment but that differed with respect to the length of the 3' overhangs (27, 54, and 80 nt) that were predicted to accommodate one or more RPA complexes. SUMO, E1, E2, Siz2, and DNA concentrations were maintained at a constant level while RPA concentration and 3' overhang length were varied. If SUMO‐RPA did not exchange or if Siz2 required modification of one RPA complex prior to modification of the second, we predicted that differences would arise in SUMO conjugation rates when comparing substrates depicted in Fig 10A. We observed that rates of SUMO modification, up to the point where 63 ± 5% of Rfa1 is modified, were independent of the ssDNA length or RPA concentration used (Fig 10A and Appendix Fig S7). These experiments are consistent with a model in which Siz2 remains bound to dsDNA, that SUMO‐RPA and RPA complexes are in dynamic exchange, and that RPA can more readily displace SUMO‐RPA, thus allowing RPA to occupy a position proximal to Siz2 to facilitate successive rounds of modification.

## Discussion

RPA‐coated single‐stranded DNA generates a platform that contributes to the hierarchical organization of factors that promote DNA repair (Maréchal & Zou, 2015). RPA post‐translational modifications can facilitate the repair response by modulating interactions between RPA and its partners (Maréchal & Zou, 2015; Dhingra *et al*, 2019). Previous studies in yeast showed that RPA and other HR proteins are modified by SUMO in a Siz2‐ and ssDNA‐dependent manner (Psakhye & Jentsch, 2012; Chung & Zhao, 2015; Dhingra *et al*, 2019). Our present results complement this model and suggest an important role for the junction between double‐ and single‐stranded DNA, at least for Siz2‐dependent SUMO conjugation of RPA. Although the structural basis for these interactions remains to be determined, we hypothesize that a DNA duplex with a 3' overhang positions RPA relative to Siz2 so that SUMO can be readily transferred to RPA. Rfa1 lysine 170 is located just upstream of the DBD‐A domain of Rfa1 (Fig 1A). Interestingly, in the crystal structure of a RPA homologue bound to single‐stranded DNA (Fan & Pavletich, 2012), the N‐terminus of the DBD‐A domain is located very close to the 5'‐end of the single‐stranded DNA. If true, this would position the Siz2/E2˜SUMO complex bound to double‐stranded DNA in the general vicinity of lysine 170 as visualized in our working model of the complex (Appendix Fig S8). Lysine 170 would then be in a preferred orientation for recognition by the E2 because of its presence in an inverted SUMO consensus site for conjugation (sequence ERKF conforming to the motif acidic‐space‐lysine‐hydrophobic). This prediction is supported by our biochemical data showing that lysine 170 is the preferred site of modification.

Our *in vitro* data underscore the importance of the Siz2 SAP domain with respect to its DNA‐binding activities. While DNA binding is clearly important for modification of RPA *in vitro*, it is less clear *in vivo* as a Siz2 variant mutated at three positions within its SAP domain (G64A K66A L69A) still supported RPA modification by SUMO under conditions of DNA damage (Chung & Zhao, 2015). We were unable to assess the activity of this mutant *in vitro* because it exhibited poor expression and solubility, a behavior also observed *in vivo* as two independent groups reported lower levels of the Siz2 mutant when expressed using the native Siz2 promoter (Psakhye & Jentsch, 2012; Chung & Zhao, 2015). Although it is likely that Siz2 DNA binding contributes to SUMO modification of RPA *in vivo*, it is also possible that other factors may guide to Siz2 to regions of DNA resection and repair.

Our results suggest that amplification of the SUMO‐RPA signal at sites of DNA damage may occur through exchange of RPA complexes adjacent to the junction between single‐stranded and double‐stranded DNA. While RPA binds DNA with considerable affinity, its interactions with DNA are highly dynamic due to the presence of multiple DNA‐binding domains (Sugitani & Chazin, 2015; Chen *et al*, 2016). Further structural and biochemical work will be required, but we envision a model (Fig 10B) where Siz2 remains bound to dsDNA proximal to the junction between double‐stranded and single‐stranded DNA where it mediates SUMO conjugation of RPA molecules to generate a pool of SUMO‐RPA complexes that can engage other repair proteins through SUMO–SIM interactions as proposed by Jentsch and colleagues (Psakhye & Jentsch, 2012; Jentsch & Psakhye, 2013). HR proteins, such as Rad51, were indeed shown to be recruited at sites of DNA damage in a SIM‐ and E3‐dependent manner, possibly through enhanced interactions with SUMO‐modified proteins (Shima *et al*, 2013). However, in the context of collapsed forks, recent evidence suggests that SUMO‐modified RPA may prevent Rad51 binding to collapsed forks prior to their translocation to the nuclear pore complex (Whalen *et al*, 2020). Thus, generating a pool of SUMO‐RPA molecules proximal to a lesion may facilitate accumulation of other HR proteins such as Rad51 while maintaining them in a paused state, ready to intervene during later stages of repair.

## Materials and Methods

### Cloning, protein expression, and purification

Expression and purification of Smt3(1–98), Smt3(19–98 K19R), Smt3(1–98 K11C D68R), and yeast E1 composed of Aos1(1–347) and Uba2(1–554) was performed as described (Mossessova & Lima, 2000; Yunus & Lima, 2009a; Streich & Lima, 2016). *Ubc9*(1–157) was cloned into the NdeI/BlpI sites of pET11c to express a native tagless protein. *Rfa1*(1–621) was cloned into the NcoI/HindIII sites of pBADHisA to express a tagless protein. *Rfa2*(1–273) and *Rfa2*(1–205) were cloned into the BamHI/NotI sites of pRSFDuet vectors to generate proteins with noncleavable N‐terminal hexahistidine tags. *Rfa3*(1–122) was cloned into the NdeI/XhoI sites of the same vectors to express a tagless protein. *Siz2*(1–726) and *Siz2*(154–420) were inserted by topo cloning into pSmt3. *Siz2*(1–420) was cloned into the BamHI/NotI sites of pTrx28, a modified pET28b‐based plasmid that allows the production of proteins as His6‐thioredoxin fusions with a TEV‐cleavage site immediately following the thioredoxin sequence. Point mutations within Ubc9, RPA, and Siz2 were generated using QuickChange site‐directed mutagenesis following the manufacturer instructions.

All recombinant proteins were produced in *E. coli* BL21 (DE3) CodonPlus RIL (Novagen). Cells were grown at 37°C in baffled flasks with Super Broth medium (Teknova) and induced by addition of isopropyl‐β‐d‐thiogalactopyranoside (IPTG) to a final concentration of 0.3 mM followed by further incubation with shaking at 18°C for 16–20 h. RPA expression was performed using bacteria transformed with both pBADHisA/Rfa1 and pRSFDuet/Rfa2/Rfa3. In this case, L‐(+)‐Arabinose was also added to a final concentration of 0.2% (*v*/*v*) for induction. In all cases, pelleted cells were suspended in lysis buffer [20 mM Tris–HCl pH 8.0, 350 mM NaCl, 2 mM beta‐mercaptoethanol], lysed by sonication, and clarified by centrifugation. Ubc9(1‐157) was purified using SP‐Sepharose (GE Healthcare) and Superdex75 (GE Healthcare) columns. For RPA, the soluble protein extract containing Rfa1, Rfa2, and Rfa3 was applied onto an Ni‐NTA column (Qiagen). After several washes, the bound recombinant proteins were eluted with a buffer containing 20 mM Tris–HCl pH 8.0, 350 mM NaCl, and 400 mM imidazole. RPA was further purified using HiTrap Heparin HP (GE Healthcare) and Superdex200 columns (GE Healthcare). For Siz2(1–727), Siz2(1–420), and Siz2(154–420), the soluble protein extract was applied onto an Ni‐NTA column (Qiagen). After several washes, the bound recombinant proteins were eluted with a buffer containing 20 mM Tris–HCl pH 8.0, 350 mM NaCl, and 400 mM imidazole. Siz2(1–727) was further purified using a HiTrap Heparin HP column. The His6‐Smt3 tag was then cleaved with the Ulp1 protease (Mossessova & Lima, 2000). Both Ulp1 and His6‐Smt3 were then removed using a Ni‐NTA column (Qiagen), and Siz2(1–727) was further purified on a Superdex200 column. Following purification on a Superdex200 column, the His6‐Smt3 tag of Siz2(154–420) was cleaved with the Ulp1 protease and both Ulp1 and His6‐Smt3 were removed using a MonoS column (GE Healthcare). For Siz2(1–420), the His6‐thioredoxin tag was cleaved with TEV. Siz2(1–420) was then further purified using a Superdex75 column. Smt3, Ubc9, RPA, and Siz2 point mutants were expressed and purified as their native counterparts. SUMO‐modified RPA was obtained from SUMO conjugation reactions conducted at 30°C in 20 mM HEPES 7.5, 125 mM NaCl, 0.1% (*v*/*v*) Tween‐20, 5 mM MgCl_2_, 2 mM ATP and 3 mM DTT with 100 nM E1∆CT, 100 nM Ubc9, 100 nM Siz2, 10 µM RPA, 1 µM ds20ss32 DNA, and 50 µM Smt3 for 35 min. The reaction mixture was then applied onto Capto HiRes S column (GE Healthcare) equilibrated with 20 mM HEPES 7.5, 50 mM NaCl, 0.1 mM DTT. The adsorbed proteins were eluted using a linear gradient of NaCl from 50 mM to 500 mM in 20 mM HEPES 7.5, 50 mM NaCl, 0.1 mM DTT. Mono‐SUMO‐modified RPA eluted at ˜270 mM NaCl. Following purification, all proteins were concentrated, flash cooled in liquid nitrogen, and stored at −80°C until needed.

### Crystallization and data collection

Microcrystals of Siz2(154–420) were obtained upon concentrating the protein by ultrafiltration in a buffer consisting of 20 mM Tris–HCl pH 8.0, 150 mM NaCl, and 1 mM beta‐mercaptoethanol. This solution served as a seeding solution to grow larger crystals that were obtained by purifying and concentrating Siz2(154–420) to 533 µM in a buffer consisting of 20 mM Tris–HCl pH 8.0, 350 mM NaCl, and 1 mM beta‐mercaptoethanol. Prior to crystallization, this protein was diluted to 186 µM with a solution consisting of 20 mM Tris–HCl pH 8.0, 100 mM NaCl, 20% glycerol, and 1 mM beta‐mercaptoethanol. 2 µl of seeding solution was then added to 100 µl of this solution. Crystals grew for 10 days in a hanging drop equilibrated against 40% PEG3350 and were flash cooled by plunging into liquid nitrogen.

### Structure determination and refinement

Data collection was performed at 100 K at the Advanced Photon Source (APS) (Argonne, IL), Northeastern Collaborative Access Team (NE‐CAT) beamline 24‐ID‐C. Indexing, integration, and scaling of the diffraction data were performed with HKL2000 (Otwinowski & Minor, 1997). Molecular replacement was performed with PHENIX (Adams *et al*, 2010) and the crystal structure of Siz1 (PDB 3I2D (Yunus & Lima, 2009b)) as a search model. Refinement and model building were performed with PHENIX (Adams *et al*, 2010) and COOT (Emsley *et al*, 2010), respectively. Figures were prepared with PyMOL (http://www.pymol.org/). The model for Siz2 SAP domain was obtained via the SWISS‐MODEL homology‐modeling server (Waterhouse *et al*, 2018).

### DNA oligomers

The following oligonucleotides (5′‐to‐3′ sequences) were purchased from Integrated DNA Technologies (IDT): ss32, TTT TTT TTT TTT TTT TTT TTT TTT TTT TTT TT; fw20, GCT GTA GCT AGT TTT GCT TC; rv20ss32, GAA GCA AAA CTA GCT ACA GCT TTT TTT TTT TTT TTT TTT TTT TTT TTT TTT T; ss32fw20, TTT TTT TTT TTT TTT TTT TTT TTT TTT TTT TTG CTG TAG CTA GTT TTG CTT C; rv20, GAA GCA AAA CTA GCT ACA GC; fb20ss27, GTA CCC GTG ACA GCT CTC CGT TTT TTT TTT TTT TTT TTT TTT TTT TT; fb20ss54, GTA CCC GTG ACA GCT CTC CGT TTT TTT TTT TTT TTT TTT TTT TTT TTT TTT TTT TTT TTT TTT TTT TTT TTT TT; fb20ss80, GTA CCC GTG ACA GCT CTC CGT TTT TTT TTT TTT TTT TTT TTT TTT TTT TTT TTT TTT TTT TTT TTT TTT TTT TTT TTT TTT TTT TTT TTT TTT TTT TTT T; fb20ss80, GTA CCC GTG ACA GCT CTC CGT TTT TTT TTT TTT TTT TTT TTT TTT TTT TTT TTT TTT TTT TTT TTT TTT TTT TTT TTT TTT TTT TTT TTT TTT TTT TTT T; rb20, CGG AGA GCT GTC ACG GGT AC; fw20Fl (6‐FAM)GCT GTA GCT AGT TTT GCT TC; fw20(P), (Phosphate)GCT GTA GCT AGT TTT GCT TC; rv20Fl (6‐FAM)GAA GCA AAA CTA GCT ACA GC; rv20(P), (Phosphate)GAA GCA AAA CTA GCT ACA GC;and ss32Fl (6‐FAM)TTT TTT TTT TTT TTT TTT TTT TTT TTT TTT TT; rv20ss32(P), (Phosphate)GAA GCA AAA CTA GCT ACA GCT TTT TTT TTT TTT TTT TTT TTT TTT TTT TTT T;. ds20, ss32ds20, ds20ss32, ds20ss27, ds20ss54, and ds20ss80 were obtained by annealing fw20 and rv20, ss32fw20 and rv20, rv20ss32 and fw20, fb20ss27 and rb20, fb20ss54 and rb20, and fb20ss80 and rb20, respectively. The oligonucleotides with single 5′ phosphate group, ds20(P)ss32, (P)ds20ss32, ss32ds20(P), were obtained by annealing rv20ss32 and fw20(P), rv20ss32(P) and fw20, and ss32fw20 and rv20(P) respectively. The 5′ fluorescein‐labeled ds20, ds20ss32, and ss32ds20 oligonucleotides used in fluorescence polarization assays were obtained by annealing rv20 and fw20Fl, rv20ss32 and fw20Fl, and ss32fw20 and rv20Fl, respectively.

### SUMO conjugation assays performed under multiple‐turnover conditions

SUMO conjugation assays presented in Figs 1, 2, 4, 6 and 7, and Appendix Fig S3 were conducted at 30°C in 20 mM HEPES 7.5, 125 mM NaCl, 0.1% (*v*/*v*) Tween‐20, 5 mM MgCl_2_ and 3 mM DTT with 100 nM E1∆CT, 100 nM Ubc9, 100 nM Siz2, 2 µM RPA or RPA‐DNA, and 50 µM Smt3. Reactions were initiated by ATP addition to a final concentration of 2 mM. At the indicated time points, aliquots were removed and quenched in NuPage LDS Sample Buffer (Life Technologies) supplemented with 106 mM 2‐mercaptoethanol. Samples were run at 180 V on 4–12% SDS–PAGE with MOPS running buffer (Life Technologies). Gels were stained with Coomassie (Bio‐Rad) or with SYPRO Ruby (Bio‐Rad) and imaged on a Typhoon 9500 (GE Healthcare) with a 473‐nm laser and an LPG filter. Initial SUMO conjugation rates were obtained by measuring the decrease in intensity of the bands corresponding to unmodified Rfa1 as a function of time using ImageJ (NIH). Initial rates were calculated from data obtained within the linear range (up to the 20‐min time‐point) and were normalized to the rate obtained with non‐mutated proteins.

SUMO conjugation assays presented in Appendix Fig S2 reactions were performed under the same conditions as assays presented in Figs 1, 2, 4, 6 and 7 except that: (*i*) RPA was labeled with Alexa488‐maleimide (Life Technologies) according to manufacturer instructions, (*ii*) unstained gels were first imaged for Alexa488 signal with a 473‐nm laser and an LPB filter using Typhoon 9500 (GE Healthcare) before SYPRO Ruby (Bio‐Rad) staining and imaging.

SUMO conjugation assays presented in Fig 10 were conducted at 30°C in 20 mM HEPES 7.5, 125 mM NaCl, 0.1% (*v*/*v*) Tween‐20, 5 mM MgCl_2_ and 3 mM DTT with 100 nM E1∆CT, 100 nM Ubc9, 1 µM Siz2, 1 µM DNA, 1 to 3 µM RPA and 50 µM Smt3. Reactions were initiated by ATP addition to a final concentration of 2 mM and processed as described previously except for the following differences: (i) To ensure that roughly the same proportion of Rfa1 would be modified by SUMO at the end of the kinetics, time‐courses were stopped at 6, 12, and 18 min when using RPA concentration of 1, 2, and 3 µM, respectively. (ii) Samples were diluted such that the final amount of RPA loaded was equivalent between lanes. Reported rates (s^−1^) were obtained by dividing measured rates (µM s^−1^) by the Ubc9 concentration (0.1 µM in all cases).

### SUMO conjugation assays under single‐turnover conditions

Smt3(1–98 K11C D68R) was labeled with Alexa488‐maleimide (Life Technologies) according to manufacturer instructions. The Smt3˜Ubc9 thioester adduct was prepared at 30°C in 20 mM HEPES, pH 7.5, 50 mM NaCl, 0.1% (*v*/*v*) Tween‐20, 5 mM MgCl_2_, 0.4 mM DTT and 2 mM ATP with 1 μM E1∆CT, 17.25 μM untagged Ubc9, and 20 μM Alexa488‐labeled Smt3(1‐98 K11C D68R). After 6 min, the reaction was passed on a MonoQ column equilibrated with 50 mM sodium citrate, pH 5.5, and 50 mM NaCl. Flow‐through fractions were pooled and injected on a MonoS column equilibrated with 50 mM sodium citrate, pH 5.5, and 50 mM NaCl. Elution was performed by applying a NaCl gradient from 50 mM to 1 M. Glycerol was added to the fractions containing the Smt3˜Ubc9 thioester adduct to a final concentration of 10%, and aliquots of this solution were flash cooled in liquid nitrogen and stored at −80°C until needed.

Assays under single‐turnover conditions were conducted at 23°C in 20 mM HEPES, pH 7.5, 125 mM NaCl and 0.1% (*v*/*v*) Tween‐20 and were initiated by addition of thioester‐charged Alexa488‐labeled Smt3(1‐98 K11C D68R)˜Ubc9(1–157 K153R) to serially diluted RPA–DNA complexes in the presence of 50 nM Siz2(1–420). At the indicated time points, aliquots were removed and rapidly quenched in a buffer containing 50 mM HEPES, pH 7.5, 2% SDS, 4 M urea, 10% glycerol, and 0.25% bromophenol blue. Samples were run at 180 V on a 4–12% SDS–PAGE with MOPS running buffer (Life Technologies). Gels were imaged on a Typhoon 9500 (GE Healthcare) with a 473‐nm laser and an LPB filter, and bands were quantified with ImageJ (NIH). Eight different RPA‐DNA concentrations were used with three time points per concentration. Experiments were performed in technical triplicates. Data were fitted to the equation *V* = *V*_max_ [S]/(*K*_d_ + [S]) in Prism 7 (GraphPad), in which *V*_max_ = k2[E]t, k2 is the rate constant, [E]t is the Smt3˜Ubc9 thioester concentration, *K*_d_ is the apparent dissociation constant, and [S] is the substrate concentration.

### Fluorescence polarization (FP)

Fluorescence polarization experiments were performed at 23°C using a SpectraMax M5 (Molecular Devices) microplate reader with excitation, emission, and cutoff wavelengths of 485, 525, and 515 nm, respectively. Measurements presented in Fig 4, Appendix Figs S5–S7 were performed with 50 nM 5′‐fluorescein‐labeled DNA substrates and serially diluted target proteins in a buffer consisting of 20 mM HEPES, pH 7.5, 125 mM NaCl, and 0.1% (*v*/*v*) Tween‐20. Measurements presented in Appendix Fig S6 were performed with 1 nM 5′‐fluorescein‐labeled ss32 DNA and serially diluted target proteins in a buffer consisting of 20 mM HEPES, pH 7.5, 125 mM NaCl, 5 mM MgCl_2_, 1 mM DTT, and 0.1% (*v*/*v*) Tween‐20. Experiments were performed in technical triplicates and analyzed in Prism 7 with a single‐site binding model accounting for ligand depletion, as previously described (Cappadocia *et al*, 2015).

### Electrophoretic mobility shift assays (EMSA)

DNA‐binding assays presented in Fig 8 were conducted using 5′ fluorescein‐labeled 32 nt poly‐T DNA probe (ss32Fl). For direct DNA‐binding reactions presented in Fig 8A, 50 nM of probe in 20 mM HEPES pH 7.5, 125 mM NaCl, 0.1% (v/v) Tween‐20, 5 mM MgCl_2_, 1 mM DTT was mixed with equal volume of 12.5, 25, 37.5, 50, 62.5, 75, or 87.5 nM or RPA or SUMO‐RPA in the same buffer, resulting in final concentrations of 25 nM DNA probe and 0–43.75 nM (0–1.75 equiv.) of RPA or SUMO‐RPA. The reactions were incubated for 25 min at 23°C. SUMO‐RPA and RPA exchange reactions presented in Fig 8B were conducted using the same conditions except RPA:DNA or SUMO‐RPA:DNA complexes, generated by preincubating 50 nM of DNA probe with 50 nM of RPA or SUMO‐RPA in 20 mM HEPES pH 7.5, 125 mM NaCl, 0.1% (v/v) Tween‐20, 5 mM MgCl_2_, 1 mM DTT, for 25 min at room temperature, were used instead of DNA. After indicated incubation times, samples were diluted with Novex Hi‐Density TBE Sample Buffer (5×) to a final concentration of 0.5× and loaded onto native 6% polyacrylamide DNA gels that were subsequently run at 90V at room temperature in 0.5× TBE buffer. Gels were imaged on a Typhoon 9500 (GE Healthcare) with a 473‐nm laser and an LPG filter and quantified by measuring intensity of the bands corresponding to RPA:DNA or SUMO‐RPA:DNA complexes.

Binding assays presented in Fig 9 were carried out using 5′ fluorescein‐labeled DNA probe (ds20ss32). First, RPA:DNA, RPA∆WH:DNA, and SUMO‐RPA:DNA complexes were obtained by incubation of 20 nM RPA variant and 20 nM DNA probe in 20 mM HEPES pH 7.5, 125 mM NaCl, 0.1% (v/v) Tween‐20, 5 mM MgCl_2_, for 25 mins at 23°C. The resulting complexes or DNA probe alone was then mixed with equal volume of serially diluted Siz2 in the same buffer. After 25 min of incubation at 23°C, samples were diluted with Novex Hi‐Density TBE Sample Buffer (5×) to a final concentration of 0.5× and loaded onto native 6% polyacrylamide DNA gels. Gels were run at 90 V at room temperature in 0.5× TBE buffer and imaged on a Typhoon 9500 (GE Healthcare) with a 473‐nm laser and an LPG filter. Siz2 binding was studied by measuring intensity of the bands corresponding to unbound DNA probe or its complex with RPA variant as a function of Siz2 concentration. Experiments were performed in technical triplicates and analyzed in Prism 7 with a single‐site binding model accounting for ligand depletion, as previously described (Cappadocia *et al*, 2015).

## Author contributions

LC, TK, and CDL conceived the study and designed experiments that LC and TK performed.

## Conflict of interest

The authors declare that they have no conflict of interest.

## Supporting information



Appendix

## Data Availability

Atomic coordinates and structure factors for Siz2 have been deposited in the Protein Data Bank (PDB) under accession codes 6U75: https://www.rcsb.org/structure/6U75.
