# Supplementary material for: DNA asymmetry promotes SUMO modification of the single‐stranded DNA‐binding protein RPA
Source: EMBO J. 2021 Sep 29;40(22):e103787. doi: 10.15252/embj.2019103787 (PMC8591536; doi:10.15252/embj.2019103787)
Supplement: Supplementary file 1 — Appendix [file EMBJ-40-e103787-s002.pdf]

Table of Contents

Appendix Table S1. X-ray crystallography data collection and refinement statistics 2

Appendix Figure S1. SUMO conjugation assays performed under multiple turnover conditions using wild-type or mutant forms of RPA, SUMO and Siz2. 3

Appendix Figure S2. SUMO conjugation assays performed under multiple turnover conditions using Alexa488-RPA and Alexa488-RPA:ds20ss32 DNA complex... 4

Appendix Figure S3. SUMO conjugation to RPA by Siz2 is dependent on 3' ssDNA overhang but independent of phosphorylated 5' ends of DNA. 5

Appendix Figure S4. SUMO conjugation assays performed under single turnover conditions using different RPA-DNA complexes. 6

Appendix Figure S5. Siz2ΔCT binding to DNA and its complexes with RPA or RPAΔWH 7

Appendix Figure S6. Binding of SUMO-RPA to ssDNA 8

Appendix Figure S7. SUMO conjugation assays performed under multiple turnover conditions using different RPA-DNA complexes. 9

Appendix Figure S8. Working model of Siz2:RPA:3'-overhang DNA complex 10

Expanded References... 11

Appendix Table S1. X-ray crystallography data collection and refinement statistics

	Siz2 (PDB 6U75)
**Data collection**[†]	
Wavelength (Å)	0.9792
Resolution range (Å)	49.0-2.63 (2.73-2.63)
Space group	$P\ 1\ 2_1\ 1$
Unit cell	
a,*b*,*c* (Å)	61.92 80.69 76.53
α,β,γ (°)	90 95.00 90
Total reflections	65431 (5431)
Unique reflections	21200 (1915)
Multiplicity	3.1 (2.8)
Completeness (%)	94.5 (86.9)
Mean I/σI	11.15 (1.69)
Wilson B-factor ($Å^2$)	44.6
R_{merge}	0.090 (0.483)
R_{meas}	0.107 (0.581)
R_{pim}	0.057 (0.316)
$CC_{1/2}$	0.995 (0.726)
CC*	0.999 (0.917)
**Refinement**[†]	
Reflections used in refinement	21188 (1915)
Reflections used in the test set	1060 (92)
R_{work}	0.218 (0.238)
R_{free}	0.263 (0.299)
CC_{work}	0.928 (0.849)
CC_{free}	0.898 (0.742)
Number of non-hydrogen atoms	4237
macromolecules	4196
ligands	2
solvent	39
Number of protein residues	522
RMS_{bonds}	0.002
RMS_{angles}	0.48
Ramachandran favored, allowed, outliers (%)	95.9 3.9 0.19
Rotomer outliers	0.0
Clashscore	2.49
Average B-factor	48.4
macromolecules	48.5
ligands	48.7
solvent	42.2

[†]Statistics calculated using Phenix; highest shell is indicated in parentheses

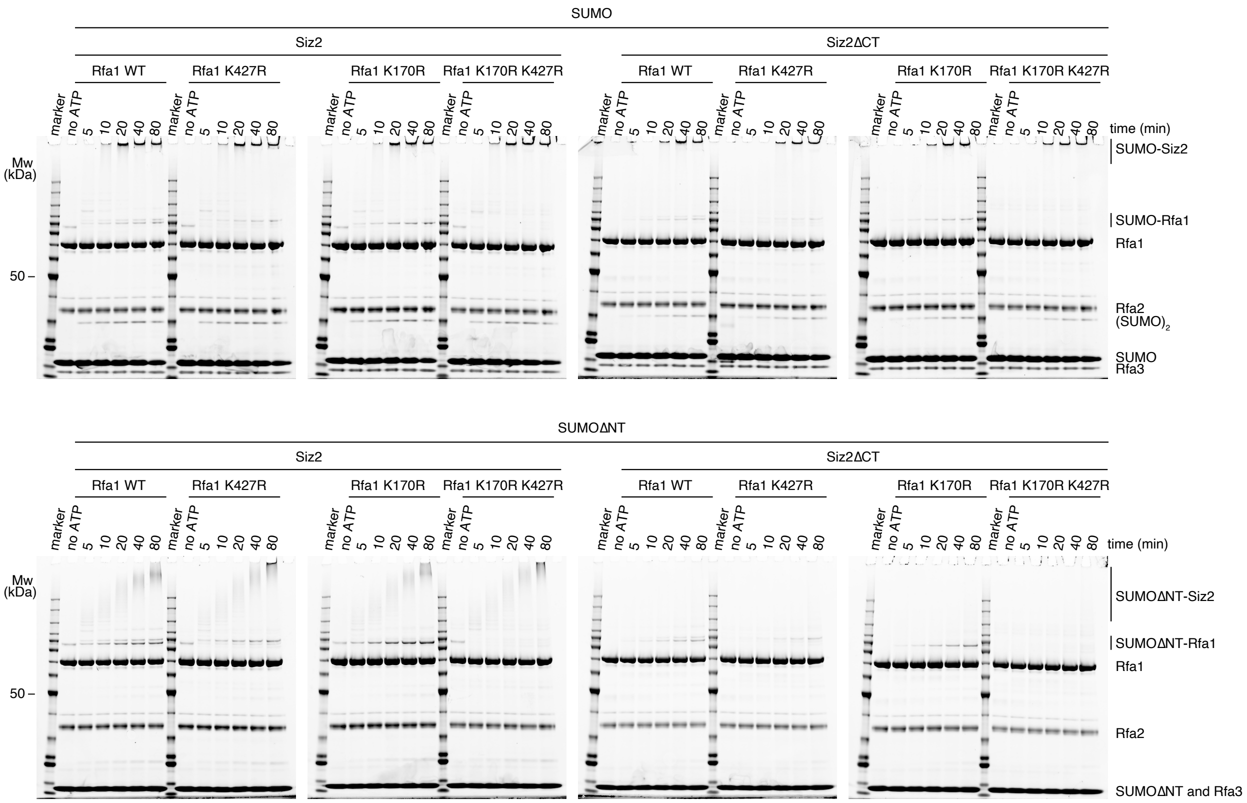

Appendix Figure S1. SUMO conjugation assays performed under multiple turnover conditions using wild-type or mutant forms of RPA, SUMO and Siz2.

Related to Fig 1. Full annotation of Mw marker bands in Figure 1B.

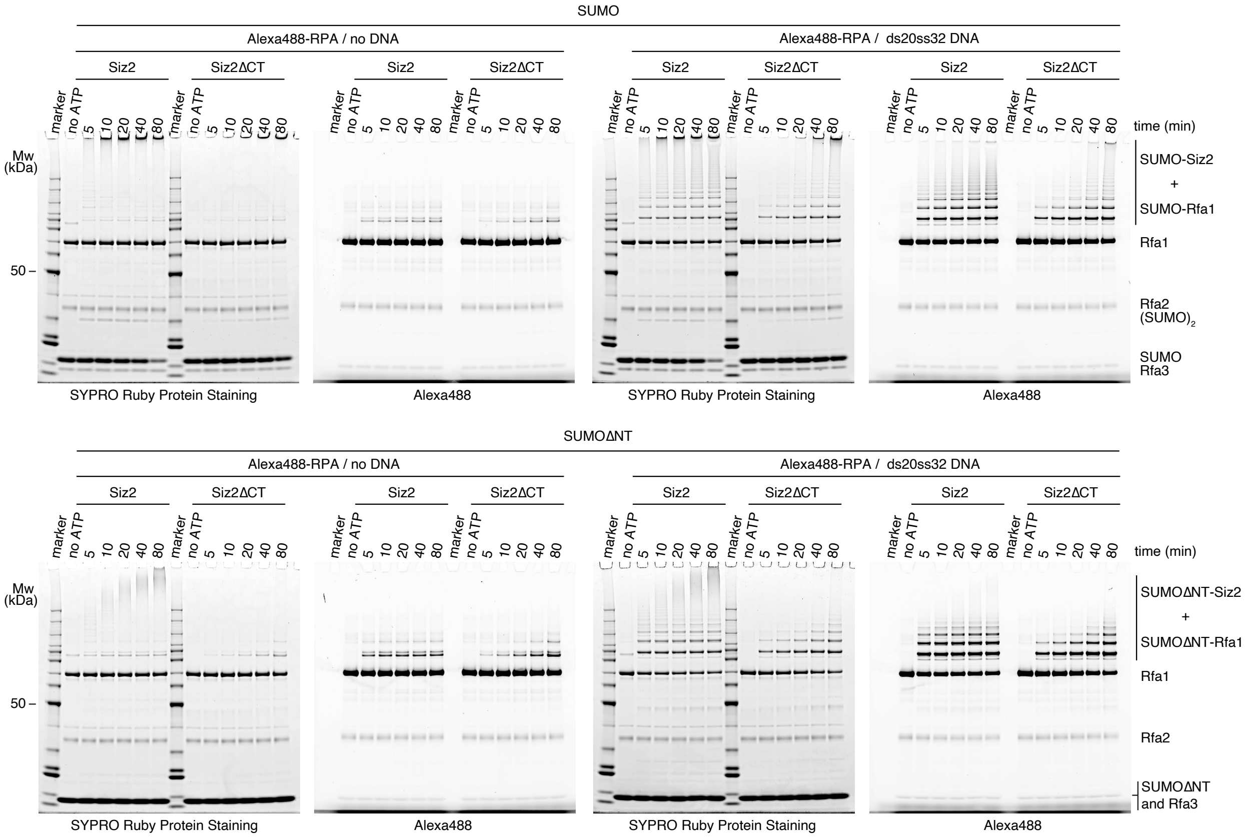

Appendix Figure S2. SUMO conjugation assays performed under multiple turnover conditions using Alexa488-RPA and Alexa488-RPA:ds20ss32 DNA complex

SDS-PAGE gels were imaged for Alexa488 signal, stained with SYPRO Ruby and imaged for SYPRO signal. Full annotation of Mw marker bands in Figure 1B.

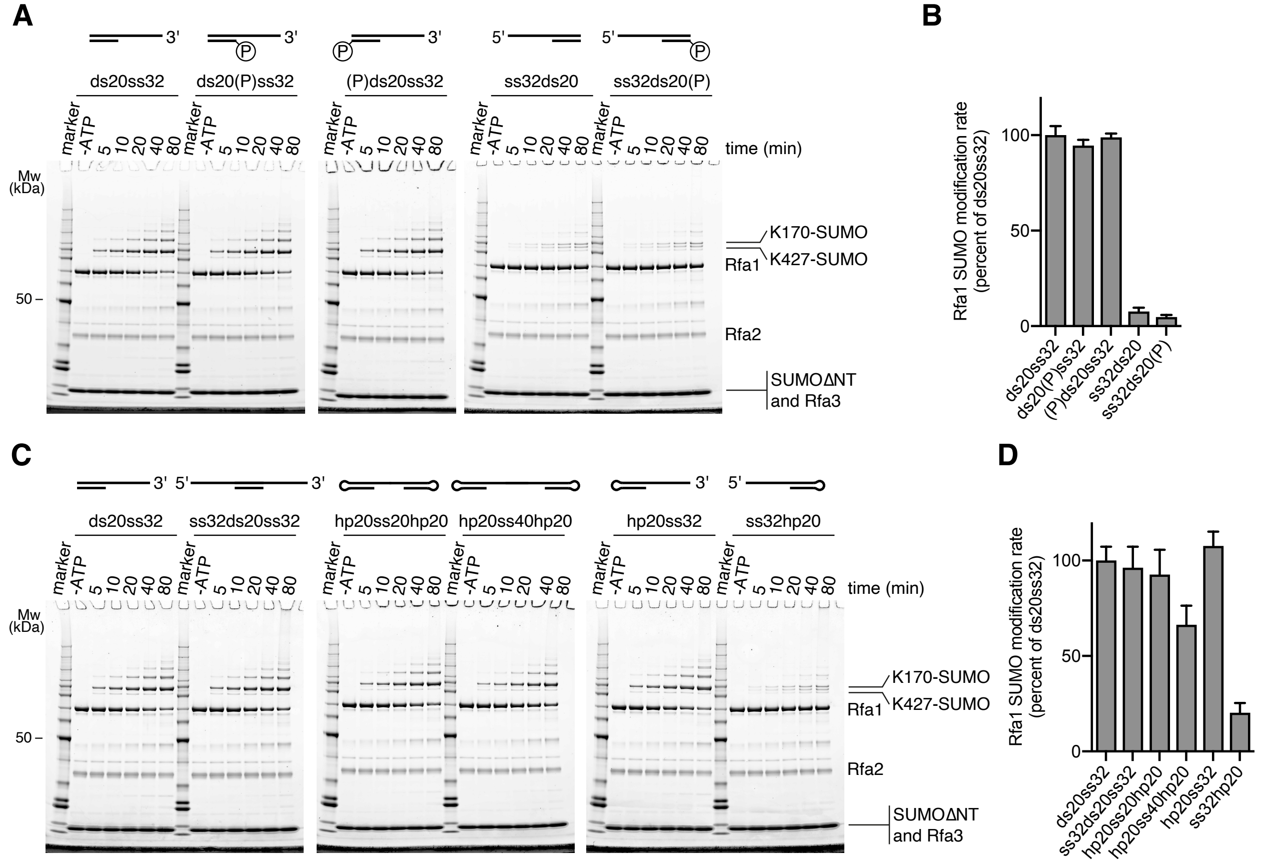

Appendix Figure S3. SUMO conjugation to RPA by Siz2 is dependent on 3' ssDNA overhang but independent of phosphorylated 5' ends of DNA.

A, C SUMO conjugation assays performed under multiple turnover conditions using Siz2ΔCT and different RPA-DNA complexes. In both cases, reactions were done in technical triplicate and SDS-PAGE gels were stained with SYPRO Ruby. Only one representative gel is shown.

B, D Histograms derived from the gels presented in (A) and (C) and presenting Rfa1 SUMO conjugation rates for RPA complexes with different DNA templates.

Data information: In (B, D) data show mean ± s.d. for three technical replicates. Full annotation of Mw marker bands in Figure 1B.

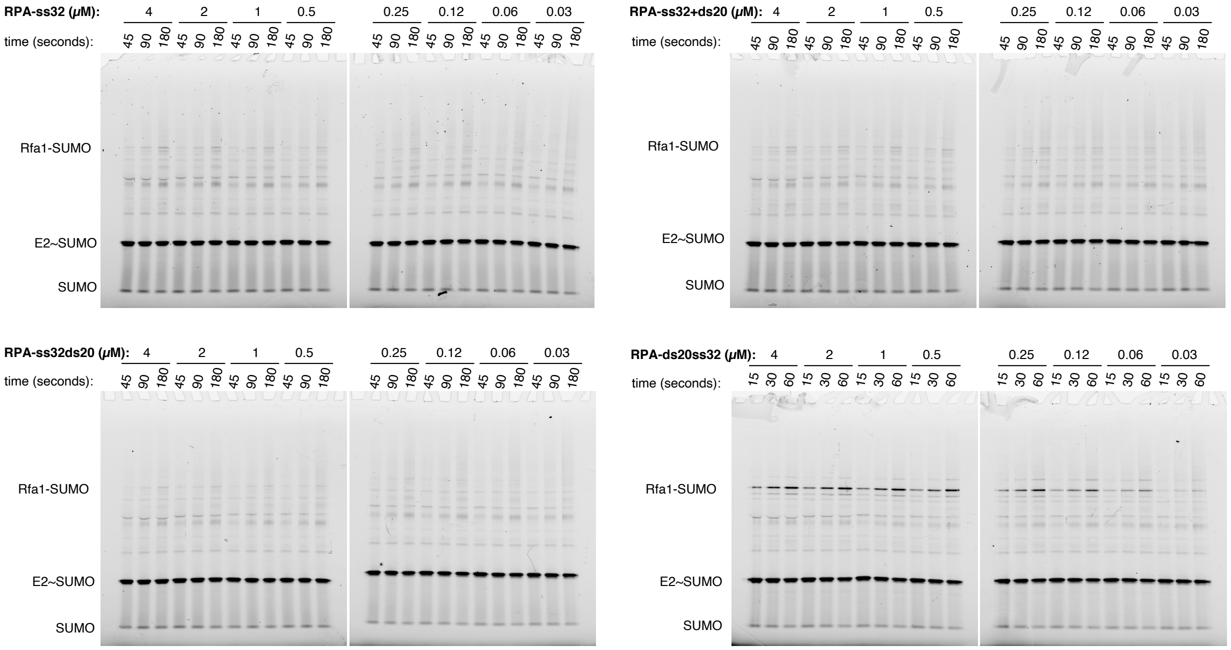

Appendix Figure S4. SUMO conjugation assays performed under single turnover conditions using different RPA-DNA complexes.

Representative SDS-PAGE gels of experiments performed in technical triplicates. Related to Fig 3.

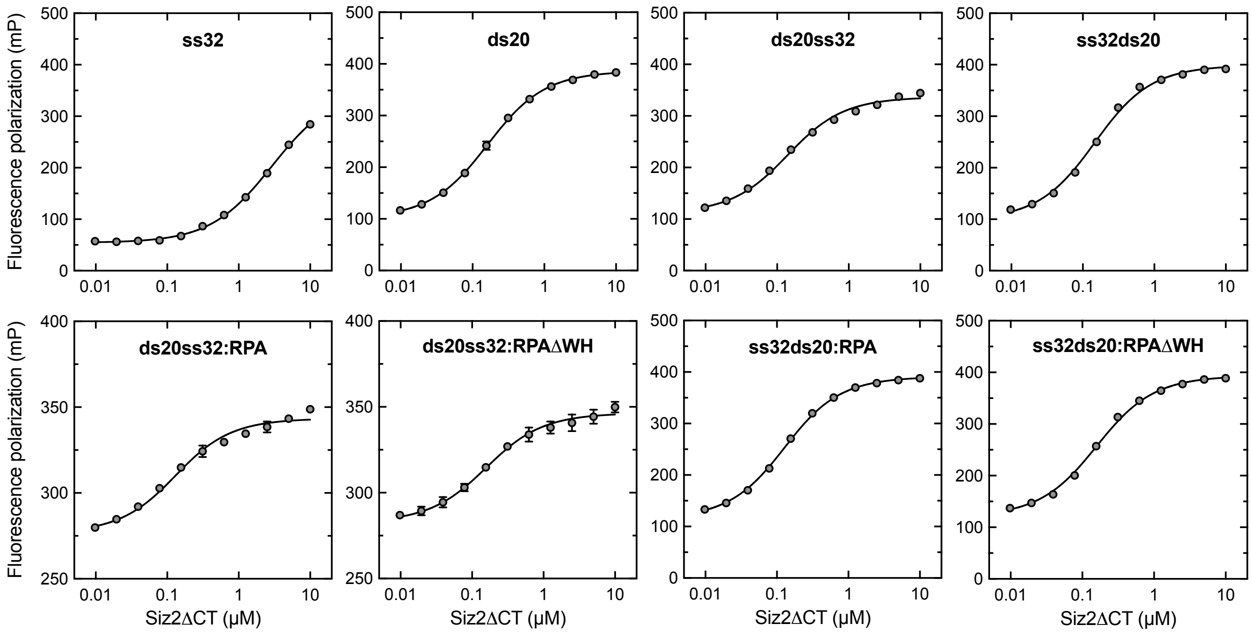

Appendix Figure S5. Siz2ΔCT binding to DNA and its complexes with RPA or RPAΔWH

Fluorescence polarization titrations performed in technical triplicates. Related to Fig 4.

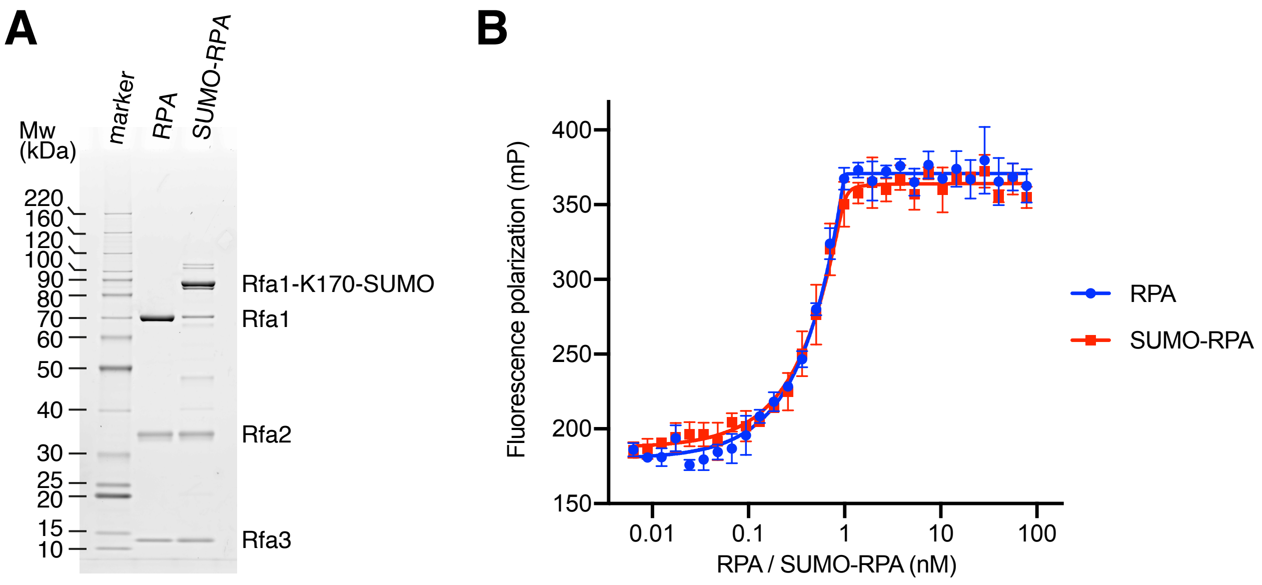

Appendix Figure S6. Binding of SUMO-RPA to ssDNA

A SYPRO-stained SDS-PAGE showing purified RPA and SUMO-RPA.

B Binding of RPA SUMO-RPA ssDNA as measured by fluorescence polarization of 1 nM 6-FAM-labeled 32dT DNA analyzed with a single-site binding model accounting for ligand depletion. Both SUMO-RPA and RPA demonstrate stoichiometric DNA binding under assay conditions. As stoichiometric binding occur when the DNA concentration is equal to or higher than the dissociation constant (Binz *et al*, 2006), these data suggest sub-nanomolar dissociation constant for DNA binding of RPA and SUMO-RPA.

Data information: In (B) data show mean ± s.d. for three technical replicates.

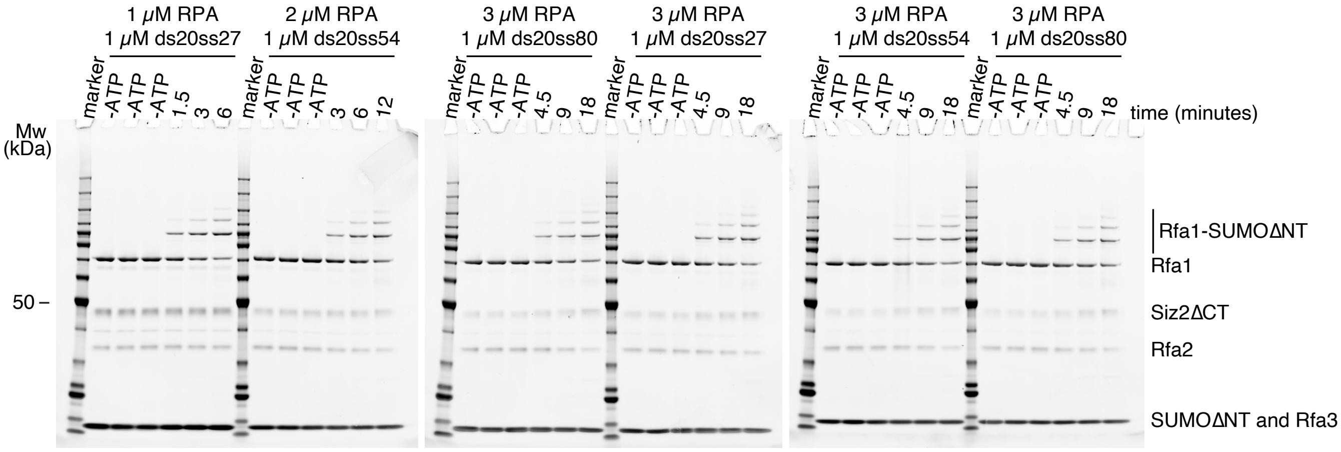

Appendix Figure S7. SUMO conjugation assays performed under multiple turnover conditions using different RPA-DNA complexes.

Representative SDS-PAGE gels stained with SYPRO of experiments performed in technical triplicates. Samples were diluted to load the same total RPA quantity in each well. At the last time point, 63 ± 5 % of Rfa1 was modified. Related to Fig 10. Full annotation of Mw marker bands in Figure 1B.

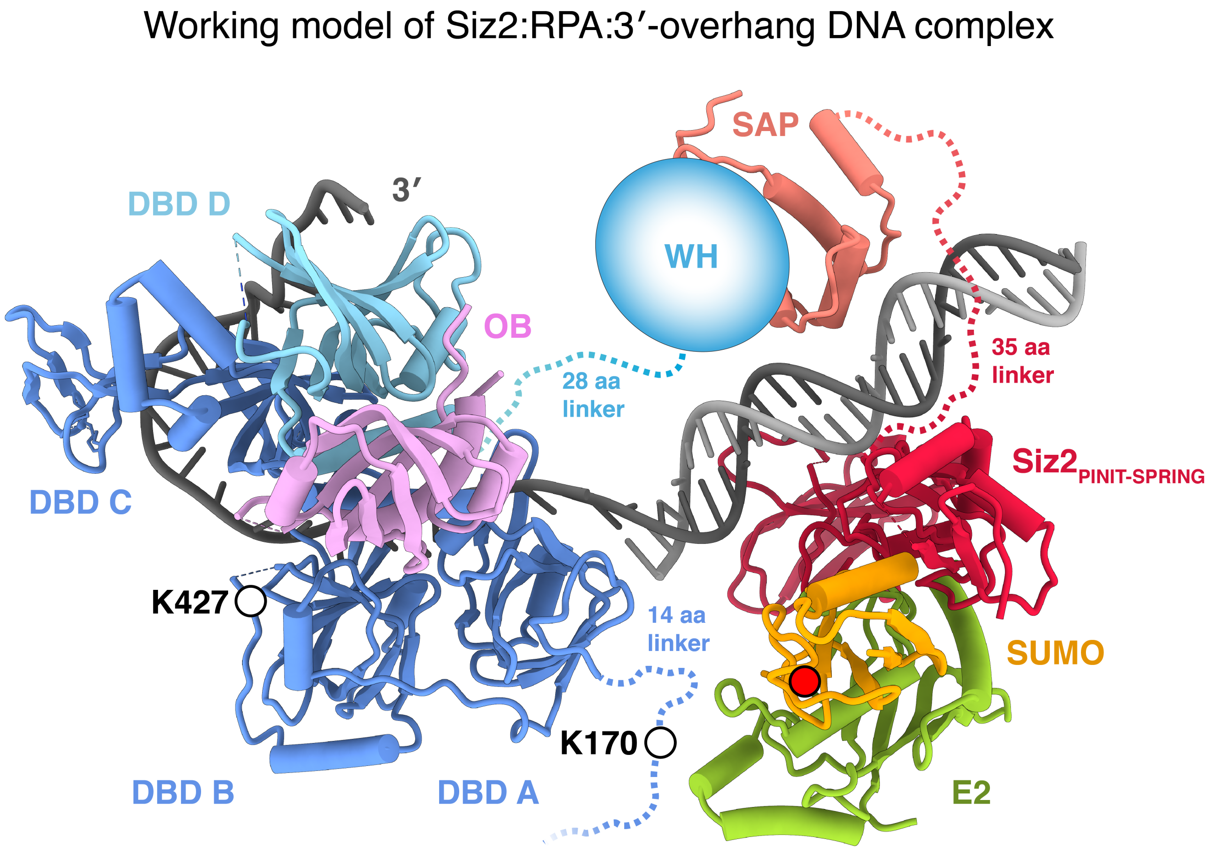

Appendix Figure S8. Working model of Siz2:RPA:3′-ssDNA overhang DNA complex

Working 3D model of a Siz2:RPA:DNA complex prior to SUMO transfer generated using a structure of the *U. maydis* RPA heterotrimer bound to ssDNA (pdb id: 4GOP) (Fan & Pavletich, 2012), a computational model of dsDNA (Arnott *et al*, 1976) with a manually docked SAP domain of Siz1 (pdb id 2RNN) (Suzuki *et al*, 2009) and the structure of Siz2$^{PINIT\text{-}SPRING}$ that is reported in this work. E2$_{Ubc9}$-SUMO^{D} is docked onto Siz2$^{PINIT\text{-}SPRING}$ based on a structure of E2$_{Ubc9}$-SUMO^{D}/E3$_{Siz1}$-SUMO^{B}/PCNA (pdb 5JNE) (Streich & Lima, 2016). Rpa70 (DBD A, DBD B, DBD C), Rpa32 (DBD D) and Rpa14 (OB) proteins forming RPA heterotrimer are shown in blue, light cyan and pink, respectively. Winged helix domain (WH) that was excluded from the crystallized RPA heterotrimer is depicted as cyan sphere. The SAP and PINIT-SPRING Siz2 domains are shown in light red and red, respectively, proximal to dsDNA. E2 and SUMO are shown in green and orange respectively. Main sites of RPA SUMO-modification of RPA and active site of E2 are depicted as white and red circles, respectively.

Expanded References

Arnott S, Campbell-Smith PJ, Chandrasekaran R (1976) In *Handbook of Biochemistry and Molecular Biology, 3rd ed Nucleic Acids--Volume II*, Fasman GP (ed) pp 411-422. Cleveland: CRC Press

Binz SK, Dickson AM, Haring SJ, Wold MS (2006) Functional assays for replication protein A (RPA). *Methods in enzymology* 409: 11-38

Fan J, Pavletich NP (2012) Structure and conformational change of a replication protein A heterotrimer bound to ssDNA. *Genes & development* 26: 2337-47

Streich FC, Jr., Lima CD (2016) Capturing a substrate in an activated RING E3/E2-SUMO complex. *Nature* 536: 304-8

Suzuki R, Shindo H, Tase A, Kikuchi Y, Shimizu M, Yamazaki T (2009) Solution structures and DNA binding properties of the N-terminal SAP domains of SUMO E3 ligases from Saccharomyces cerevisiae and Oryza sativa. *Proteins* 75: 336-47
