# Supplementary material for: DNA asymmetry promotes SUMO modification of the single‐stranded DNA‐binding protein RPA
Source: EMBO J. 2021 Sep 29;40(22):e103787. doi: 10.15252/embj.2019103787 (PMC8591536; doi:10.15252/embj.2019103787)
Supplement: Supplementary file 2 [file EMBJ-40-e103787-s001.pdf]

THE EMBO JOURNAL

DNA asymmetry promotes SUMO modification of the single-stranded DNA binding protein RPA

Laurent Cappadocia, Tomasz Kochańczyk, and Christopher Lima
DOI: 10.15252/embj.2019103787

Corresponding author(s): Christopher Lima (limac@mskcc.org)

Review Timeline:

Submission Date:	19th Oct 19
Editorial Decision:	28th Nov 19
Revision Received:	23rd Jun 21
Editorial Decision:	19th Aug 21
Revision Received:	11th Sep 21
Accepted:	14th Sep 21

Editor: Hartmut Vodermaier

Transaction Report:

(Note: With the exception of the correction of typographical or spelling errors that could be a source of ambiguity, letters and reports are not edited. Depending on transfer agreements, referee reports obtained elsewhere may or may not be included in this compilation. Referee reports are anonymous unless the Referee chooses to sign their reports.)

1st Editorial Decision 28th Nov 2019

Thank you for submitting your manuscript for our editorial considerations, and apologies for the delay in getting back to you with a decision, which was owed to limited availability and slow response of referees at that time. We have now finally received a complete set of reports from three reviewers, copied below for your information.

As you will see, although the referees acknowledge (to varying degrees) the potential interest of your biochemical findings on Siz2-RPA interplay, the considerable number of queries raised by all of them forces me to conclude that the study is at present not yet sufficiently insightful to immediately warrant EMBO Journal publication. That said, I realize that the comments include several constructive suggestions, whose incorporation and follow-up could make an extended study a significantly stronger candidate for an EMBO Journal article.

Although we usually avoid asking for major extensions of unclear outcome, but only invite revisions for manuscripts that are already quite close to becoming acceptable within a reasonable time-frame, I would in the present case (given the interest expressed by referees 2 and 3) remain open to considering a revised version of the manuscript further, in case you should be prepared and able to take on the additional mechanism-clarifying experiments asked by all three referees. I realize that this may require considerable further time and effort, but feel that such more definitive understanding would really be needed for this mainly biochemical/structural study to become suitable for our broad general journal. If required, I'd be happy to discuss a possible extension of the default three-months revision deadline, during which publication of any competing/related work would as usual not have a negative impact on our final assessment of your own study. Alternatively, I would of course also be open to discussing rapid publication of a less-extensively revised manuscript with the editors of our partner journal Life Science Alliance, in case you should be interested in such possibility. In any case, please do not hesitate to contact me already during the early stages of revision if you should have any questions regarding the reviewer comments or your plans for how to address them.

Referee #1:

In this study, the authors used purified proteins and an in vitro assay to show that 3' ssDNA overhang stimulates the SUMOylation of RPA by Siz2. The SAP domain of Siz2 and the WH domain of Rfa2 are important for RPA SUMOylation. The DNA binding of Siz2 correlates its activity in RPA SUMOylation. Using DNA structures with 3' overhangs of different lengths, they suggest that Siz2 SUMOylates RPA at ssDNA/dsDNA junctions and SUMO-RPA and RPA undergo exchange to amplify SUMO-RPA signals. The biochemical experiments in this study are well done. However, the mechanism underlying the stimulation of RPA SUMOylation by 3' overhang is not clearly explained. More importantly, the functional significance of the in vitro observations is quite unclear. This manuscript is more suitable for biochemistry-focused journal than a general journal like the EMBO J.

Specific comments:

1. In figure 2, is RPA SUMOylation stimulated by the 3' ssDNA overhang or the 5' ssDNA/dsDNA junction? It is important to distinguish the contributions of these structural components experimentally.

2. Is RPA SUMOylation affected by ssDNA gaps? This experiment should be tried and mechanistically explained. This is an important question about the structural specificity of the model.

3. Is the increase of K170 SUMOylation functionally more important than the reduction in K427 SUMOylation? Addressing this issue is important for evaluating the relevance of the in vitro stimulation of RPA SUMOylation by 3' overhang.

4. The depiction of RPA-ss32+ds20 in figure 3A is incorrect. ss32 is missing.

5. In figure 3B, why did RPA-ss32+ds20 have a lower Kd than RPA-ss32? Did ds20 affect the binding of RPA to ss32?

6. The data in figure 7A suggest that the SUMO-RPA and RPA at ssDNA/dsDNA are in dynamic exchange. However, why this exchange is specific to 5' ssDNA/dsDNA but not 3' ssDNA/dsDNA junctions is not explained. This is another important question about the structural specificity of the model.

7. Is there any evidence supporting that SUMO-RPA can reassociate with ssDNA after being released from the ssDNA/dsDNA junctions? Without this evidence, the model cannot explain how SUMO-RPA accumulates at DNA damage sites.

Referee #2:

Cappadocia and Lima show that the SUMO E3 ligase Siz2 from budding yeast carries out DNA dependent SUMO modification of the single stranded DNA binding protein RPA. As these proteins

have important roles in the repair of double stranded DNA breaks the authors tested a number of DNA templates and found that templates with a short stretch of double stranded DNA flanked by a 3' single stranded extension, that mimics end resected DNA, was most efficient at promoting DNA dependent SUMO modification of RPA.
Specific points.
1. In the in vitro assays it is clear that both Siz2 and SUMO are undergoing extensive modification, but it is not clear if Rfa1 is also being incorporated into these higher molecular weight complexes. Experiments with fluorescent Rfa1 or Western blotting should resolve this point. I can certainly see the value of using versions of Siz2 and SUMO that don't undergo SUMO modification - it will make the quantitation and determination of reaction parameters more straightforward, but such extensive modification of Siz2, SUMO and possible Rfa1 could be required for the function of SUMO in vivo.
2. In figure 2 it is very difficult to judge from the gels shown which residue the SUMO is going on to. In the text it is indicated that it is predominantly K170 but looking at the figure I can't see how this conclusion is reached. In the far right hand panel there is a predominant modified species, but there is also a more slowly migrating species. Does this represent both sites occupied or is this two SUMOs on K170 ? Some mass spec analysis of these products would be informative.
3. In the final figure of the paper (fig 7) the authors test two different models for "amplification of the SUMO RPA signal". What the experiment in Fig 7 demonstrates is that SUMO modification of RPA does not alter its DNA binding properties and thus once modified it can dissociate from the template and be replaced by an unmodified molecule. I don't think this says anything about amplification of the SUMO signal. The difficulty is that we do not know what SUMO modification of RPA does in vivo. It could be that SUMO modification allows the RPA to be retained at sites of DNA damage by interactions between the SUMO and multiple components of the DNA repair machinery.
4. While the experiments reported in the paper show a clear role for the SAP domain in recruitment of the E3 ligase to DNA, there does not seem to be a role for the SIM region of Siz2. I wonder if this might be revealed by doing direct DNA binding assays with DNA template bound to either RPA or SUMO modified RPA and monitoring recruitment of Siz2 to the template.
In general, I think this is an interesting paper with nicely done biochemistry that illustrates an important principle.

Referee #3:

Overall Summary

The authors examine the biochemical and structural basis for SUMO modification of Replication Protein A (RPA) by DNA damage SUMO E3 ligase Siz2 from budding yeast. They demonstrate that RPA SUMOylation is stimulated by ds/ssDNA substrates with 3' overhangs, is dependent upon DNA-binding by Siz2's SAP domain, and is enhanced by an intact RPA32C protein interaction domain (winged helix domain). Their structure of Siz2's catalytic PINIT/SP-RING domains exhibits high similarity to related DNA-dependent SUMO E3 ligase Siz1, which acts on PCNA. They propose an exchange mechanism for signal propagation of SUMOylated RPA on damage-resected ssDNA, where dynamic release of SUMOylated RPA from the ds/ssDNA junction allows non-modified RPA to bind and encounter the Siz2 SUMOylation platform.

The expanding universe of post-translational modifications involved in DNA damage signaling and repair coordination (SUMOylation, ubiquitination, poly(ADP-ribosylation), etc) makes this work timely and of broad interest. The ability of damage relevant DNA substrates to regulate a DNA damage

signaling SUMO E3 ligase makes this work particularly exciting.

The presented work itself is overall technically sound. The redundancy of the crystallographic data is on the low side (~3), but the R-factors for the model of the Siz2 catalytic core are in line with the resolution of the dataset.

General Comments:

While the authors' biochemistry experiments convincingly demonstrate that interactions from DNA substrates and RPA regulate Siz2, the structural investigations of Siz2 do not address how Siz2-DNA-RPA interactions might promote/regulate RPA SUMOylation. Instead, their structural analyses focus upon general comparisons between the domain fold of the Siz2 catalytic core and the related SUMO E3 ligase Siz1. This is interesting, but does not illuminate the regulatory mechanisms uncovered by the biochemical investigations. The impact and novelty of this study could be greatly strengthened by focusing the structural analyses on the Siz2-SAP domain and DNA/RPA interactions. This would, ideally, generate a testable model of Siz2-DNA-RPA assembly on resection substrates and provide greater insight into propagation of RPA SUMOylation signaling.

Some key points that could be considered:

• How does the SAP domain engage DNA? Is its preferred substrate ds/ssDNA junctions with 3' overhangs? Does RPA impact substrate preference or affinity?

• The study alludes to a potential interaction between the Siz2-SAP domain and the RPA32C domain that could explain enhanced RPA SUMOylation when the RPA32C domain is present. There is a known RPA32C interaction motif in human RPA (Feldkamp, 2014). Does this motif exist in yeast Siz2-SAP? The availability of the authors' SAP domain homology model and known RPA32C substrate complexes offers the possibility of constructing a homology model of the complex for subsequent testing.

• Are the RPA SUMOylation sites (R170, R427) and the RPA32C domain the only RPA sites of putative interaction with Siz2?

• It is intriguing that both DNA binding by the Siz2-SAP domain and its proposed interactions with RPA32C enhance Siz2 SUMOylation, as these points of contact should, in theory, occur on opposite sides of RPA's DNA binding site (SAP DNA contacts near the 5' end of the binding site, RPA32C contacts near the 3' end of the binding site). Can Siz2-SAP simultaneously contact the ds/ssDNA junction and RPA32C? Do simultaneous or mutually exclusive complexes impact efficient RPA SUMOylation?

• Regarding the catalytic PINIT/SP-RING domains - there is a marked contrast between the rigid, well-defined structure of the Siz1 PCNA substrate and the disordered RPA linker surrounding K170 that is targeted by Siz2. Can the authors comment on whether there are unique structural features of Siz2 PINIT/SP-RING that enable recognition of the SUMOylation site in this disordered context? Is there a Siz SUMOylation motif that is common to RPA and other Siz substrates?

A model for Siz2-DNA-RPA assembly could also provide hypotheses for more rigorous testing of the exchange model of RPA group modification by Siz2. The authors conclude that intrinsic RPA exchange dynamics enable propagation of the SUMOylation signal from a Siz2 platform at the ds/ssDNA junction. Could RPA exchange be facilitated? - either by reduced affinity for DNA when

SUMOylated or through interactions between RPA domains and Siz2? Other RPA protein partners are proposed to displace RPA from ssDNA through interactions with the RPA32C domain (Arunkumar, 2005, NSMB). A Siz2-driven displacement of RPA from its catalytic platform would be an attractive and exciting model to explain propagation of the SUMOylation signal.

Specific Edits/Recommendations:

p. 6, 2nd paragraph: The references for UNG2 (Mer et al, 2000) and SMARCAL1 (Feldkamp et al, 2014) are reversed.

p. 7, 2nd paragraph: Which specific Siz2 residues are important for contacting the E2 and SUMO? Which non-conserved residues contribute compensating interactions, and what are these interactions? The residues at these interfaces should be displayed in a zoomed view, so that readers can assess these conclusions for themselves. Given the lower resolution of the Siz2 catalytic domain structure and the surface location of these residues, it is understandable that side chain positioning may be uncertain, but a comparison of the interfaces would be helpful.

Figure 4f: The authors should explain how 'similar' or 'different' residues are defined in their surface mapping. Ideally, a quantitative measure of conservation should be used (as provided by UCSF Chimera's conservation rendering feature with sequence alignments).

We thank the reviewers for their comments and insightful suggestions. In response, we conducted several new studies. The following new experimental data is now presented in the form of new figures and discussed in the revised manuscript:

- SUMO conjugation assays with fluorescently labeled RPA to differentiate SUMO modified RPA from other SUMO conjugation products (Figure EV2).
- SUMO conjugation assays using RPA in the presence of DNA substrates with 5'-phosphate end to evaluate the influence of phosphorylated DNA end on conjugation (Figures EV3A and EV3B).
- SUMO conjugation assays using RPA in the presence of DNA substrates with 3'- and 5'- overhang hairpin to evaluate the contribution of dsDNA ends to conjugation, 20-nt and 40-nt gap dumbbell and double overhang DNA templates to evaluate DNA substrates that include ssDNA gaps (Figures EV3C and EV3D).
- Binding studies between Siz2 and various substrates to analyze the contribution of DNA, RPA and the RPA-WH domain to these interactions (Figures 4D and EV5).
- Characterization of DNA binding constants for purified SUMO-RPA for comparison to RPA (Figure EV6).
- Competitive binding studies to determine the relative abilities of RPA or SUMO-RPA to compete for binding DNA substrates (Figure 8).
- Binding studies using full-length Siz2 and SUMO-RPA or RPA bound to DNA to determine if SUMO-interaction motifs in full-length Siz2 contribute to binding (Figure 9).

Referee #1:

In this study, the authors used purified proteins and an in vitro assay to show that 3' ssDNA overhang stimulates the SUMOylation of RPA by Siz2. The SAP domain of Siz2 and the WH domain of Rfa2 are important for RPA SUMOylation. The DNA binding of Siz2 correlates its activity in RPA SUMOylation. Using DNA structures with 3' overhangs of different lengths, they suggest that Siz2 SUMOylates RPA at ssDNA/dsDNA junctions and SUMO-RPA and RPA undergo exchange to amplify SUMO-RPA signals. The biochemical experiments in this study are well done. However, the mechanism underlying the stimulation of RPA SUMOylation by 3' overhang is not clearly explained. More importantly, the functional significance of the in vitro observations is quite unclear. This manuscript is more suitable for biochemistry-focused journal than a general journal like the EMBO J.

We thank the reviewer for the expert synopsis and hope that our revisions satisfy their major concerns.

Specific comments:

1. In figure 2, is RPA SUMOylation stimulated by the 3' ssDNA overhang or the 5' ssDNA/dsDNA junction? It is important to distinguish the contributions of these structural components experimentally.

In addition to several binding studies described below, we now include SUMO modification of RPA with additional substrates that preserve the 3' overhang but remove the 5'ssDNA/dsDNA junction by placing the duplex region of the substrate at an internal position and by creating two substrates with a hairpin at the 3' or 5' end (Figures EV3C and EV3D). Importantly, Siz2 binds to all DNA substrates containing a duplex DNA segment, but only DNA substrates that contain a 3'-overhang display higher rates of SUMO-modification of RPA, and these rates appear independent of the 5' end configuration. To further probe structural determinants required for productive RPA SUMO-modification, we also examined the effect of the presence of phosphate group at 5' end of DNA on the SUMO-modification (Figures EV3A and EV3B). We did not observe differences in SUMO-modification, suggesting that stimulation of SUMO-modification is not dependent on the configuration of the 5'-end. While we cannot fully exclude the possibility that 5' dsDNA end at the junction contributes to recognition, additional binding studies (Figures 4B and EV5) suggests that Siz2 binds equally well to duplex substrates lacking a 5' dsDNA end and that stimulation of SUMO conjugation to RPA by Siz2 is consistent with the orientation of RPA when bound to the 3'-overhang (see next comment).

2. Is RPA SUMOylation affected by ssDNA gaps? This experiment should be tried and mechanistically explained. This is an important question about the structural specificity of the model.

We now include assays to evaluate SUMO modification of RPA in the presence of substrates with a 20-nt or 40-nt ssDNA gap buttressed by hairpins on the respective ends (Figure EV3C and EV3D). Similar rates of SUMO modification were observed for these substrates compared to other substrates with 3' overhangs.

Taken together, data presented in Figure EV3 in SI and discussed on page 5 in the manuscript suggest that stimulation of SUMO modification of RPA by Siz2 depends on DNA substrates that include dsDNA with a 3'-overhang but is independent of DNA end configuration. These data are in agreement with our proposed model where the Siz2 SAP domain binds dsDNA to position it proximal the 3' ssDNA where RPA is then bound in an optimal orientation for modification of K170. In the proposed model the proximity between K170 and the duplex moiety is based on (i) the DNA polarity in the Ustilago maydis RPA:DNA crystal structure (Fan et al., Genes Dev. 2012) and (ii) the possible location of K170 in that structure. Indeed, K170 is located 15 residues upstream of DBD A, a domain that contacts the 5' end of the ssDNA. In the structure of Ustilago maydis RPA in complex with ssDNA, the first ordered residue of DBD A is located 24 Å away from the 5' end of the ssDNA (where the DNA duplex will be in the 3'-overhang configuration) whereas it is 69 Å away from the 3' end (where the DNA duplex will be in the 5'-overhang configuration). See panel A and B in figure below for models with dsDNA with 3'-overhang and 5'-overhang respectively. The differences would be even greater if the RPA protomer were extended relative to the U-shape seen below.

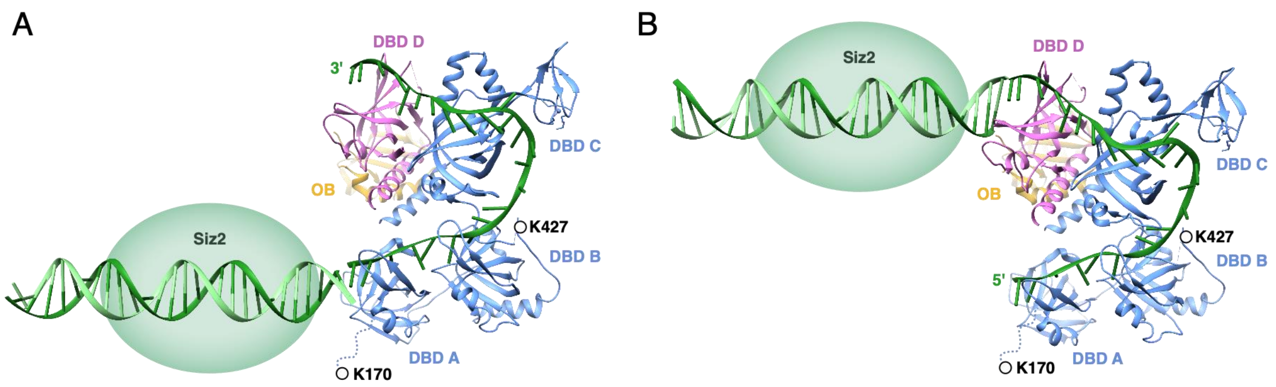

3. Is the increase of K170 SUMOylation functionally more important than the reduction in K427 SUMOylation? Addressing this issue is important for evaluating the relevance of the in vitro stimulation of RPA SUMOylation by 3' overhang.

We do not believe that one site is more important than another based on genetic evidence provided by Psakhye and Jentsch as mutational studies showed that their individual mutation does not disrupt DNA repair, while defects were observed when added collectively in the context of additional mutations in other repair factors (Psakhye et al., Cell 2012). This is consistent with the protein group modification hypothesis as presented in their paper. Furthermore, Zhao and colleagues had to mutate both positions in addition to two minor sites (K180 and K411) to uncover a phenotype for SUMO-RPA in the DNA damage checkpoint (Dhingra et al., J. Biol. Chem. 2019). We added additional comments on page 10 to discuss this point in more detail.

4. The depiction of RPA-ss32+ds20 in figure 3A is incorrect. ss32 is missing.

This error has been corrected.

5. In figure 3B, why did RPA-ss32+ds20 have a lower Kd than RPA-ss32? Did ds20 affect the binding of RPA to ss32?

We can only speculate why it is lower. In unpublished work we noted that isolated SAP domain can inhibit DNA-dependent SUMO conjugation if added in trans, but that inhibition can be alleviated by adding more duplex DNA suggesting that in the absence of DNA the SAP domain may interact with other domains in Siz2 to either inhibit its interaction with substrate or with the charged E2. Since the K_d is less than 3-fold lower and does not contribute to an increase in rate or the specificity constant, we prefer not to speculate on this point further.

6. The data in figure 7A suggest that the SUMO-RPA and RPA at ssDNA/dsDNA are in dynamic exchange. However, why this exchange is specific to 5' ssDNA/dsDNA but not 3' ssDNA/dsDNA junctions is not explained. This is another important question about the structural specificity of the model.

We now include data in Figure 8 showing that SUMO-RPA and RPA can exchange on ssDNA. While both exhibit nM binding affinities in direct DNA binding experiments (Figures EV6 and 8), SUMO-RPA is more readily displaced by RPA. The reviewer asks why this is specific to 5' ssDNA/dsDNA but not 3' ssDNA/dsDNA junctions. We believe it is specific because SUMO modification is promoted when RPA is bound to a 3' overhang, and this does not occur on DNA substrates lacking 3' overhangs. We infer that the SAP domain binds dsDNA to facilitate modification of the proximal RPA because rates of modification appear independent of the length of ssDNA (Figure 10A). We revised the model somewhat based on new data (reviewer point 5).

7. Is there any evidence supporting that SUMO-RPA can reassociate with ssDNA after being released from the ssDNA/dsDNA junctions? Without this evidence, the model cannot explain how SUMO-RPA accumulates at DNA damage sites.

In response to this and other reviewer comments below, we purified SUMO-RPA and used it to measure affinities to various substrates as shown in Figures EV8, 8 and 9. These new data are discussed on manuscript page 8. To answer directly, yes, SUMO-RPA can re-associate with ssDNA after being released from the ssDNA/dsDNA substrate.

Referee #2:

Cappadocia and Lima show that the SUMO E3 ligase Siz2 from budding yeast carries out DNA dependent SUMO modification of the single stranded DNA binding protein RPA. As these proteins have important roles in the repair of double stranded DNA breaks the authors tested a number of DNA templates and found that templates with a short stretch of double stranded DNA flanked by a 3' single stranded extension, that mimics end resected DNA, was most efficient at promoting DNA dependent SUMO modification of RPA.

We thank the reviewer for their concise and accurate summary.

Specific points.

1. In the in vitro assays it is clear that both Siz2 and SUMO are undergoing extensive modification, but it is not clear if Rfa1 is also being incorporated into these higher molecular weight complexes. Experiments with fluorescent Rfa1 or Western blotting should resolve this point. I can certainly see the value of using versions of Siz2 and SUMO that don't undergo SUMO modification - it will make the quantitation and determination of reaction parameters more straightforward, but such extensive modification of Siz2, SUMO and possible Rfa1 could be required for the function of SUMO in vivo.

We conducted studies using RPA fluorescently labeled with Alexa488 and determined that the high molecular weight species are not RPA, but can be attributed to auto-modified Siz2 or SUMO chains (Figure EV2).

2. In figure 2 it is very difficult to judge from the gels shown which residue the SUMO is going on to. In the text it is indicated that it is predominantly K170 but looking at the figure I can't see how this conclusion is reached. In the far right hand panel there is a predominant modified species, but there is also a more slowly migrating species. Does this represent both sites occupied or is this two SUMOs on K170? Some mass spec analysis of these products would be informative.

With respect to the first point, the conclusion of K170 based on the mutational data presented in Figure 1 as well as data in Zhao and colleagues (Dhingra et al., J. Biol. Chem. 2019 – Figure 1E therein) that shows a similar pattern. We also infer that the higher molecular weight species represents SUMO at both sites as an extended time course with either mutant shows reduced levels of the higher molecular weight species (Figure EV1). Furthermore, Zhao and colleagues uncovered these major sites in addition to two minor sites (K180 and K411) (Dhingra et al., J. Biol. Chem. 2019). As K170 and K427 have been validated in a variety of studies, since our biochemical data is consistent with these data, and since the exact site of modification does not matter in vivo, we do not believe that mass spec analysis is warranted given our mutational confirmation as presented in Figure 1.

3. In the final figure of the paper (fig 7) the authors test two different models for "amplification of the SUMO RPA signal". What the experiment in Fig 7 demonstrates is that SUMO modification of RPA does not alter its DNA binding properties and thus once modified it can dissociate from the template and be replaced by an unmodified molecule. I don't think this says anything about amplification of the SUMO signal. The difficulty is that we do not know what SUMO modification of RPA does in vivo. It could be that SUMO modification allows the RPA to be retained at sites of DNA damage by interactions between the SUMO and multiple components of the DNA repair machinery.

In conjunction with the ability of SUMO-RPA to re-associate with ssDNA and based on new data provided in Figures EV6 and 8 where binding affinities, exchange and stability were assessed, we believe that these data are consistent with the model as revised. While the function for these individual modifications is perhaps not so clear, we know that SUMO-RPA has a role in repair (Psakhye et al., Cell 2012), signaling (Dhingra et al., J. Biol. Chem. 2019) and recruitment (Bonner et al., Cell Reports 2016), but is likely independent of the exact site of modification. Consistent with this concept, redundancy is also observed for yeast PCNA where modification on either K164 (major site) or K127 (minor site) is sufficient to recruit the anti-recombinogenic helicase Srs2 (Pfander et al., Nature 2005; Papouli et al., Mol Cell 2005; Armstrong et al., Nature 2012).

4. While the experiments reported in the paper show a clear role for the SAP domain in recruitment of the E3 ligase to DNA, there does not seem to be a role for the SIM region of Siz2. I wonder if this might be revealed by doing direct DNA binding assays with DNA template bound to either RPA or SUMO modified RPA and monitoring recruitment of Siz2 to the template.

We conducted binding studies to directly compare affinities of full-length Siz2 (containing the SIM region) to RPA or SUMO-RPA in Figure 9. We were unable to measure a significant contribution of the SIM or SUMO to binding with these samples, although it remains possible that SIMs contribute to formation of high molecular weight species as discussed in response to this reviewer's first point (EV2).

In general, I think this is an interesting paper with nicely done biochemistry that illustrates an important principle.

We are very grateful for these comments and hope that the added revisions solidify this opinion.

Referee #3:

Overall Summary

The authors examine the biochemical and structural basis for SUMO modification of Replication Protein A (RPA) by DNA damage SUMO E3 ligase Siz2 from budding yeast. They demonstrate that RPA SUMOylation is stimulated by ds/ssDNA substrates with 3' overhangs, is dependent upon DNA-binding by Siz2's SAP domain, and is enhanced by an intact RPA32C protein interaction domain (winged helix domain). Their structure of Siz2's catalytic PINIT/SP-RING domains exhibits high similarity to related DNA-dependent SUMO E3 ligase Siz1, which acts on PCNA. They propose an exchange mechanism for signal propagation of SUMOylated RPA on damage-resected ssDNA, where dynamic release of SUMOylated RPA from the ds/ssDNA junction allows non-modified RPA to bind and encounter the Siz2 SUMOylation platform.

The expanding universe of post-translational modifications involved in DNA damage signaling and repair coordination (SUMOylation, ubiquitination, poly(ADP-ribosylation), etc) makes this work timely and of broad interest. The ability of damage relevant DNA substrates to regulate a DNA damage signaling SUMO E3 ligase makes this work particularly exciting.

The presented work itself is overall technically sound. The redundancy of the crystallographic data is on the low side (~3), but the R-factors for the model of the Siz2 catalytic core are in line with the resolution of the dataset.

We thank the reviewer for their thoughtful synopsis and comments.

General Comments:

While the authors' biochemistry experiments convincingly demonstrate that interactions from DNA substrates and RPA regulate Siz2, the structural investigations of Siz2 do not address how Siz2-DNA-RPA interactions might promote/regulate RPA SUMOylation. Instead, their structural analyses focus upon general comparisons between the domain fold of the Siz2 catalytic core and the related SUMO E3 ligase Siz1. This is interesting, but does not illuminate the regulatory mechanisms uncovered by the biochemical investigations. The impact and novelty of this study could be greatly strengthened by focusing the structural analyses on the Siz2-SAP domain and DNA/RPA interactions. This would, ideally, generate a testable model of Siz2-DNA-RPA assembly on resection substrates and provide greater insight into propagation of RPA SUMOylation signaling.

Some key points that could be considered:

• How does the SAP domain engage DNA? Is its preferred substrate ds/ssDNA junctions with 3' overhangs? Does RPA impact substrate preference or affinity?

We are unable to precisely answer the first question as there are no available structures of SUMO ligase SAP domains bound to DNA. The only structural information thus far comes from chemical shift perturbations (Suzuki et al., Proteins, 2009) and mutational studies (Chung et al., Genes & Development, 2015) including ours that map to the surface shown in Figure 6. To answer the second and third questions and to assess substrate specificities we present new binding studies with Siz2, RPA and DNA in Figure 4D and EV5. These data show that Siz2 binds with similar affinities to all substrates that contain a duplex region, that it binds weakly to ssDNA, and that dsDNA binding affinities are independent of the DNA substrate including those with ssDNA overhangs 5' or 3' of the duplex.

• The study alludes to a potential interaction between the Siz2-SAP domain and the RPA32C domain that could explain enhanced RPA SUMOylation when the RPA32C domain is present. There is a known RPA32C interaction motif in human RPA (Feldkamp, 2014). Does this motif exist in yeast Siz2-SAP? The availability of the authors' SAP domain homology model and known RPA32C substrate complexes offers the possibility of constructing a homology model of the complex for subsequent testing.

We attempted to generate different models for WH-SAP interactions based on available structures and models, but none were convincing. In addition, we could not identify Siz2 sequences with high similarity to known RPA32C target interaction motifs (Feldkamp et al., Biochemistry 2014, Mer et al, Cell 2000).

• Are the RPA SUMOylation sites (R170, R427) and the RPA32C domain the only RPA sites of putative interaction with Siz2?

These two sites are the predominant sites as assessed in our study and in vivo (Psakhye et al., Cell 2012 and Dhingra et al., J. Biol. Chem. 2019) as their mutation substantially disrupts SUMO modification of this complex. With that said, Zhao and colleagues uncovered additional minor sites in (Dhingra et al., J. Biol. Chem. 2019) that yielded defects in the DNA damage response when combined with mutations at 170 and 427. It is important to note that K170 and K427 are E2-dependent in that they adhere to SUMO E2 consensus motifs for modification (hydrophobic-lysine-space-acidic) or the SUMO E2 inverted consensus motif (acidic-space-lysine-hydrophobic). As such, we believe the interaction with the RPA-DNA substrate and charged E2 are dependent on Siz2 while interaction with K170 and K427 are more likely dependent on direct interaction with the E2 as configured in this complex.

• It is intriguing that both DNA binding by the Siz2-SAP domain and its proposed interactions with RPA32C enhance Siz2 SUMOylation, as these points of contact should, in theory, occur on opposite sides of RPA's DNA binding site (SAP DNA contacts near the 5' end of the binding site, RPA32C contacts near the 3' end of the binding site). Can Siz2-SAP simultaneously contact the ds/ssDNA junction and RPA32C? Do simultaneous or mutually exclusive complexes impact efficient RPA SUMOylation?

In some models, the RPA32C domain could contact the SAP domain of Siz2 given the linker length between SAP and PINIT-SPRING domains in Siz2 (20 amino acids) and the linker between the OB fold and RPA32C domain of Rfa2 (50 amino acids). Since deletion (not mutations) of the WH domain does not decrease Siz2 binding affinity to RPA-DNA complexes (Figures 4, EV5 and 9), we cannot determine the individual contributions of SAP-DNA or SAP-WH to binding, or whether they can occur simultaneously.

• Regarding the catalytic PINIT/SP-RING domains - there is a marked contrast between the rigid, well-defined structure of the Siz1 PCNA substrate and the disordered RPA linker surrounding K170 that is targeted by Siz2. Can the authors comment on whether there are unique structural features of Siz2 PINIT/SP-RING that enable recognition of the SUMOylation site in this disordered context? Is there a Siz SUMOylation motif that is common to RPA and other Siz substrates?

As stated earlier in response to this reviewer, both of K170 and K427 are E2-dependent in that they adhere to the SUMO E2 consensus motif (hydrophobic-lysine-space-acidic) or inverted consensus motif (acidic-space-lysine-hydrophobic) for modification. This is dissimilar from the Siz1-PCNA interaction that forces the E2 to modify a non-consensus site (PCNA K164), but perhaps similar to modification of PCNA K127. We revised the text on page 10 to include information that K170 is present within an inverted SUMOylation consensus site (sequence ERKF conforming to the motif acidic-space-lysine-hydrophobic).

A model for Siz2-DNA-RPA assembly could also provide hypotheses for more rigorous testing of the exchange model of RPA group modification by Siz2. The authors conclude that intrinsic RPA exchange dynamics enable propagation of the SUMOylation signal from a Siz2 platform at the ds/ssDNA junction. Could RPA exchange be facilitated? - either by reduced affinity for DNA when SUMOylated or through interactions between RPA domains and Siz2? Other RPA protein partners are proposed to displace RPA from ssDNA through interactions with the RPA32C domain (Arunkumar, 2005, NSMB). A Siz2-driven displacement of RPA from its catalytic platform would be an attractive and exciting model to explain propagation of the SUMOylation signal.

As stated in earlier sections, we conducted additional studies to analyze binding affinities, modification, and exchange, each of which support our general model that SUMO-RPA can be displaced by free RPA and that SUMO-RPA can re-associate with ssDNA after modification (or presumably when it's the predominant species). We also observed that SUMO-RPA is more readily displaced by RPA.

We attempted to further substantiate our model by conducting a series of UV protein DNA cross-linking studies using a series of 15 unique 61-mer dsDNA substrates with 32 nucleotide 3' overhangs containing single BrdU substitutions every 5-nt in one strand of the duplex. Unfortunately, and despite significant expense and effort, these studies were inconclusive due to non-specific crosslinking or crosslinking that could not be attributed to any particular combination of protein factors.

Specific Edits/Recommendations:

p. 6, 2nd paragraph: The references for UNG2 (Mer et al, 2000) and SMARCAL1 (Feldkamp et al, 2014) are

reversed.

This has been corrected.

p. 7, 2nd paragraph: Which specific Siz2 residues are important for contacting the E2 and SUMO? Which non-conserved residues contribute compensating interactions, and what are these interactions? The residues at these interfaces should be displayed in a zoomed view, so that readers can assess these conclusions for themselves. Given the lower resolution of the Siz2 catalytic domain structure and the surface location of these residues, it is understandable that side chain positioning may be uncertain, but a comparison of the interfaces would be helpful.

We added panels to show the interface in more detail as presented in Figure 5.

Figure 4f: The authors should explain how 'similar' or 'different' residues are defined in their surface mapping. Ideally, a quantitative measure of conservation should be used (as provided by UCSF Chimera's conservation rendering feature with sequence alignments).

We removed panel F from Figure 4. Figure 5C now shows sequence alignment and Figures 5C and 5D now show residues that are important for contacting the E2 and SUMO in Siz1 and corresponding residues in Siz2.

1st Revision - Editorial Decision 19th Aug 2021

Thank you for resubmitting a revised version of your earlier manuscript on Siz2-RPA interactions for our editorial consideration. I am very sorry for the delay associated with its re-evaluation, but as it had been quite some time since the original submission, I needed a chance to first re-assess the study myself before returning it to the referees that had initially reviewed it. Having now heard back from all three of them, I am happy to inform you that they unanimously consider the work significantly improved and would now be supportive of publication in The EMBO Journal.

I would therefore like to invite you to prepare a re-revised manuscript, incorporating the remaining specific suggestions of the reviewers (copied below), as well as the following editorial points:

Referee #1:

The authors have improved the manuscript significantly. It is acceptable for publication in the EMBO J.

Referee #2:

I think the authors have done a good job of responding to the points that I raised and am happy that the manuscript is now suitable for publication in EMBO Journal.

Referee #3:

Summary / General Comments

The authors investigate the biochemical and structural basis for SUMO modification of Replication Protein A (RPA) by the DNA damage SUMO E3 ligase Siz2 from budding yeast. They demonstrate that RPA SUMOylation is stimulated by assembly of Siz2 and RPA on ds/ssDNA substrates with 3' overhangs and propose that the Siz2 SAP domain localizes the SUMO E3 apparatus (with SUMO-E2 enzyme) to the Rfa1-170 SUMOylation site by binding dsDNA at the overhang junction. They also test and outline a mechanism for propagating RPA SUMOylation through dynamic exchange of modified for unmodified RPA at the Siz2 SUMOylation platform.

The additional biochemistry data provided by the authors strengthen and clarify the uniqueness of the 3' overhang substrate and the role for SAP-dsDNA binding in stimulating RPA SUMOylation by Siz2 (Figure 4B, EV5, EV3). The authors have also provided greater support for their model of a single SUMOylation center propagating multiple RPA SUMOylation events through dynamic exchange of modified for unmodified RPA (Figure 8 and 9). The time and research investment in this work are notable, particularly in light of the demands and restrictions imposed by the COVID-19

pandemic.

It would be helpful to have a modeled visualization of the Siz2 (SAP-PINIT-SP-RING) - RPA SUMOylation center to accompany the description in the first paragraph of the discussion. A starting point is provided by the Siz2-RPA-DNA overhang figure provided in the response to Reviewer #1, which could be enhanced with the authors crystal structure of the PINIT/SP-RING domains and SAP domain homology model. Such a visualization would be useful in synthesizing the mechanistic implications of the biochemistry.

The discussion could also benefit from exploring the implications of the dissociative model of SUMO-RPA amplification on DNA repair. RPA binding to ssDNA substrates can be dynamic, but dynamic dissociation is usually coupled to interactions between RPA's protein interaction domains (RPA70N, RPA32C) and other DNA repair factors as part of a coordinated hand-off mechanism of the ssDNA substrate. One would also assume that SUMOylating RPA in situ on the ssDNA substrate would be preferred, both to maintain protection of the ssDNA and to retain the damage modification/signal near the site of damage (i.e. on the resected ssDNA). Is there a purpose/benefit to creating a pool of SUMOylated RPA that is dissociated from the substrate? Are there specific repair factors (particularly in the HR pathway) that rely on SIMs and could use these domains to recruit or displace SUMO-RPA?

Specific suggestions:

p. 5 '...it was independent of a 5'-phosphate...'

We thank the reviewers for their comments and suggestions. The revised manuscript contains following changes and additions:

- Added visualization of a putative model of SUMO-transfer Siz2:RPA:3′-overhang DNA complex to accompany the first paragraph of the discussion (Figure S8).
- Changed representation of error bars in panel C in figure 9 from s.d to s.e.m. so the errors for mean Kd values are reported the same way as in all figures. The conclusions remain unchanged.
- The last paragraph of the discussion has been expanded to discuss implications of SUMO modification of RPA on recruitment of HR factors.

Referee #1:

The authors have improved the manuscript significantly. It is acceptable for publication in the EMBO J.

We thank the reviewer for their careful review and positive comments.

Referee #2:

I think the authors have done a good job of responding to the points that I raised and am happy that the manuscript is now suitable for publication in EMBO Journal.

We thank the reviewer for their comments and are pleased that our revision satisfied their major concerns.

Referee #3:

Summary / General Comments

The authors investigate the biochemical and structural basis for SUMO modification of Replication Protein A (RPA) by the DNA damage SUMO E3 ligase Siz2 from budding yeast. They demonstrate that RPA SUMOylation is stimulated by assembly of Siz2 and RPA on ds/ssDNA substrates with 3' overhangs and propose that the Siz2 SAP domain localizes the SUMO E3 apparatus (with SUMO-E2 enzyme) to the Rfa1-170 SUMOylation site by binding dsDNA at the overhang junction. They also test and outline a mechanism for propagating RPA SUMOylation through dynamic exchange of modified for unmodified RPA at the Siz2 SUMOylation platform.

The additional biochemistry data provided by the authors strengthen and clarify the uniqueness of the 3' overhang substrate and the role for SAP-dsDNA binding in stimulating RPA SUMOylation by Siz2 (Figure 4B, EV5, EV3). The authors have also provided greater support for their model of a single SUMOylation center propagating multiple RPA SUMOylation events through dynamic exchange of modified for unmodified RPA (Figure 8 and 9). The time and research investment in this work are notable, particularly in light of the demands and restrictions imposed by the COVID-19 pandemic.

We thank the reviewer for their thoughtful synopsis and comments and hope that the changes included in the revised manuscript solidify this opinion.

It would be helpful to have a modeled visualization of the Siz2 (SAP-PINIT-SP-RING) - RPA SUMOylation center to accompany the description in the first paragraph of the discussion. A starting point is provided by the Siz2-RPA-DNA overhang figure provided in the response to Reviewer #1, which could be enhanced with the authors crystal structure of the PINIT/SP-RING domains and SAP domain homology model. Such a visualization would be useful in synthesizing the mechanistic implications of the biochemistry.

We now include a putative model of SUMO-transfer Siz2:RPA:3′-overhang DNA complex (Figure S8) and refer to it in the first paragraph of the discussion.

The discussion could also benefit from exploring the implications of the dissociative model of SUMO-RPA amplification on DNA repair. RPA binding to ssDNA substrates can be dynamic, but dynamic dissociation is usually coupled to interactions between RPA's protein interaction domains (RPA70N, RPA32C) and other DNA repair factors as part of a coordinated hand-off mechanism of the ssDNA substrate. One would also assume that SUMOylating RPA in situ on the ssDNA substrate would be preferred, both to maintain protection of the ssDNA and to retain the damage modification/signal near the site of damage (i.e. on the resected ssDNA). Is there a purpose/benefit to creating a pool of SUMOylated RPA that is dissociated from the substrate? Are there specific repair factors (particularly in the HR pathway) that rely on SIMs and could use these domains to recruit or displace SUMO-RPA?

The last paragraph of the discussion has been expanded to further discuss these implications and two additional references were added.

Specific suggestions:

p. 5 '...it was independent of a 5'-phosphate...'

This error has been corrected.

2nd Revision - Editorial Decision 14th Sep 2021

Thank you for submitting your final revised manuscript for our consideration. I am pleased to inform you that we have now accepted it for publication in The EMBO Journal.

EMBO PRESS

YOU MUST COMPLETE ALL CELLS WITH A PINK BACKGROUND

PLEASE NOTE THAT THIS CHECKLIST WILL BE PUBLISHED ALONGSIDE YOUR PAPER

Corresponding Author Name: Christopher D. Lima
Journal Submitted to: EMBO Journal
Manuscript Number: EMBOJ-2019-103787R

Reporting Checklist For Life Sciences Articles (Rev. June 2017)

This checklist is used to ensure good reporting standards and to improve the reproducibility of published results. These guidelines are consistent with the Principles and Guidelines for Reporting Preclinical Research issued by the NIH in 2014. Please follow the journal's authorship guidelines in preparing your manuscript.

A- Figures
1. Data
The data shown in figures should satisfy the following conditions:

- the data were obtained and processed according to the field's best practice and are presented to reflect the results of the experiments in an accurate and unbiased manner.
- figure panels include only data points, measurements or observations that can be compared to each other in a scientifically meaningful way.
- graphs include clearly labeled error bars for independent experiments and sample sizes. Unless justified, error bars should not be shown for technical replicates.
- if n< 5, the individual data points from each experiment should be plotted and any statistical test employed should be justified
- Source Data should be included to report the data underlying graphs. Please follow the guidelines set out in the author ship guidelines on Data Presentation.

2. Captions
Each figure caption should contain the following information, for each panel where they are relevant:

- a specification of the experimental system investigated (eg cell line, species name).
- the assay(s) and method(s) used to carry out the reported observations and measurements
- an explicit mention of the biological and chemical entity(ies) that are being measured.
- an explicit mention of the biological and chemical entity(ies) that are altered/varied/perturbed in a controlled manner.
- the exact sample size (n) for each experimental group/condition, given as a number, not a range;
- a description of the sample collection allowing the reader to understand whether the samples represent technical or biological replicates (including how many animals, litters, cultures, etc.).
- a statement of how many times the experiment shown was independently replicated in the laboratory.
- definitions of statistical methods and measures:
  - common tests, such as t-test (please specify whether paired vs. unpaired), simple χ2 tests, Wilcoxon and Mann-Whitney tests, can be unambiguously identified by name only, but more complex techniques should be described in the methods section;
  - are tests one-sided or two-sided?
  - are there adjustments for multiple comparisons?
  - exact statistical test results, e.g., P values = x but not P values < x;
  - definition of 'center values' as median or average;
  - definition of error bars as s.d. or s.e.m.

Any descriptions too long for the figure legend should be included in the methods section and/or with the source data.

In the pink boxes below, please ensure that the answers to the following questions are reported in the manuscript itself. Every question should be answered. If the question is not relevant to your research, please write NA (non applicable). We encourage you to include a specific subsection in the methods section for statistics, reagents, animal models and human subjects.

USEFUL LINKS FOR COMPLETING THIS FORM

http://www.antibodypedia.com
http://1degreebio.org
http://www.equator-network.org/reporting-guidelines/improving-bioscience-research-repor
http://grants.nih.gov/grants/olaw/olaw.htm
http://www.mrc.ac.uk/Ourresearch/Ethicsresearchguidance/Useofanimals/index.htm
http://ClinicalTrials.gov
http://www.consort-statement.org
http://www.consort-statement.org/checklists/view/32-consort/66-title
http://www.equator-network.org/reporting-guidelines/reporting-recommendations-for-tumo
http://datadryad.org
http://figshare.com
http://www.ncbi.nlm.nih.gov/gap
http://www.ebi.ac.uk/ega
http://biomodels.net/
http://biomodels.net/miriam/
http://jjj.biochem.sun.ac.za
http://oba.od.nih.gov/biosecurity/biosecurity_documents.html
http://www.selectagents.gov/

B- Statistics and general methods

Please fill out these boxes (Do not worry if you cannot see all your text once you press return)

1.a. How was the sample size chosen to ensure adequate power to detect a pre-specified effect size?	No statistical methods were applied to pre-determine sample size. Biochemical experiments were performed at least in technical triplicates.
1.b. For animal studies, include a statement about sample size estimate even if no statistical methods were used.	No animals were used in this study. NA
2. Describe inclusion/exclusion criteria if samples or animals were excluded from the analysis. Were the criteria pre-established?	No samples were excluded from the analysis
3. Were any steps taken to minimize the effects of subjective bias when allocating animals/samples to treatment (e.g. randomization procedure)? If yes, please describe.	This study did not allocate animals/samples to treatment. NA
For animal studies, include a statement about randomization even if no randomization was used.	NA
4.a. Were any steps taken to minimize the effects of subjective bias during group allocation or/and when assessing results (e.g. blinding of the investigator)? If yes please describe.	No blinding was used.
4.b. For animal studies, include a statement about blinding even if no blinding was done	NA
5. For every figure, are statistical tests justified as appropriate?	No statistical tests were used.
Do the data meet the assumptions of the tests (e.g., normal distribution)? Describe any methods used to assess it.	NA

Is there an estimate of variation within each group of data?	NA
Is the variance similar between the groups that are being statistically compared?	NA

C- Reagents

6. To show that antibodies were profiled for use in the system under study (assay and species), provide a citation, catalog number and/or clone number, supplementary information or reference to an antibody validation profile. e.g., Antibodypedia (see link list at top right), 1DegreeBio (see link list at top right).	No antibodies were used in this study.
7. Identify the source of cell lines and report if they were recently authenticated (e.g., by STR profiling) and tested for mycoplasma contamination.	No cell-based assays were used in this study.

* for all hyperlinks, please see the table at the top right of the document

D- Animal Models

8. Report species, strain, gender, age of animals and genetic modification status where applicable. Please detail housing and husbandry conditions and the source of animals.	NA
9. For experiments involving live vertebrates, include a statement of compliance with ethical regulations and identify the committee(s) approving the experiments.	NA
10. We recommend consulting the ARRIVE guidelines (see link list at top right) (PLoS Biol. 8(6), e1000412, 2010) to ensure that other relevant aspects of animal studies are adequately reported. See author guidelines, under 'Reporting Guidelines'. See also: NIH (see link list at top right) and MRC (see link list at top right) recommendations. Please confirm compliance.	NA

E- Human Subjects

11. Identify the committee(s) approving the study protocol.	No human subject was used in this study. NA
12. Include a statement confirming that informed consent was obtained from all subjects and that the experiments conformed to the principles set out in the WMA Declaration of Helsinki and the Department of Health and Human Services Belmont Report.	NA
13. For publication of patient photos, include a statement confirming that consent to publish was obtained.	NA
14. Report any restrictions on the availability (and/or on the use) of human data or samples.	NA
15. Report the clinical trial registration number (at ClinicalTrials.gov or equivalent), where applicable.	NA
16. For phase II and III randomized controlled trials, please refer to the CONSORT flow diagram (see link list at top right) and submit the CONSORT checklist (see link list at top right) with your submission. See author guidelines, under 'Reporting Guidelines'. Please confirm you have submitted this list.	NA
17. For tumor marker prognostic studies, we recommend that you follow the REMARK reporting guidelines (see link list at top right). See author guidelines, under 'Reporting Guidelines'. Please confirm you have followed these guidelines.	NA

F- Data Accessibility

18: Provide a "Data Availability" section at the end of the Materials & Methods, listing the accession codes for data generated in this study and deposited in a public database (e.g. RNA-Seq data: Gene Expression Omnibus GSE39462, Proteomics data: PRIDE PXD000208 etc.) Please refer to our author guidelines for 'Data Deposition'.<br><br>Data deposition in a public repository is mandatory for:<br>a. Protein, DNA and RNA sequences<br>b. Macromolecular structures<br>c. Crystallographic data for small molecules<br>d. Functional genomics data<br>e. Proteomics and molecular interactions	A data availability section is present at the end of the Materials & Methods. Atomic coordinates and structure factors for Siz2 are deposited in the Protein Data Bank under accession codes PDB 6U75.
19. Deposition is strongly recommended for any datasets that are central and integral to the study; please consider the journal's data policy. If no structured public repository exists for a given data type, we encourage the provision of datasets in the manuscript as a Supplementary Document (see author guidelines under 'Expanded View' or in unstructured repositories such as Dryad (see link list at top right) or Figshare (see link list at top right).	NA
20. Access to human clinical and genomic datasets should be provided with as few restrictions as possible while respecting ethical obligations to the patients and relevant medical and legal issues. If practically possible and compatible with the individual consent agreement used in the study, such data should be deposited in one of the major public access-controlled repositories such as dbGAP (see link list at top right) or EGA (see link list at top right).	NA
21. Computational models that are central and integral to a study should be shared without restrictions and provided in a machine-readable form. The relevant accession numbers or links should be provided. When possible, standardized format (SBML, CellML) should be used instead of scripts (e.g. MATLAB). Authors are strongly encouraged to follow the MIRIAM guidelines (see link list at top right) and deposit their model in a public database such as Biomodels (see link list at top right) or JWS Online (see link list at top right). If computer source code is provided with the paper, it should be deposited in a public repository or included in supplementary information.	NA

G- Dual use research of concern

22. Could your study fall under dual use research restrictions? Please check biosecurity documents (see link list at top right) and list of select agents and toxins (APHIS/CDC) (see link list at top right). According to our biosecurity guidelines, provide a statement only if it could.	NA
